# Xylem Cell Wall Formation in Pioneer Roots and Stems of *Populus trichocarpa* (Torr. & Gray)

**DOI:** 10.3389/fpls.2019.01419

**Published:** 2019-11-12

**Authors:** Katarzyna Marzec-Schmidt, Agnieszka Ludwików, Natalia Wojciechowska, Anna Kasprowicz-Maluśki, Joanna Mucha, Agnieszka Bagniewska-Zadworna

**Affiliations:** ^1^Department of General Botany, Faculty of Biology, Institute of Experimental Biology, Adam Mickiewicz University, Poznań, Poland; ^2^Department of Biotechnology, Faculty of Biology, Institute of Molecular Biology and Biotechnology, Adam Mickiewicz University, Poznań, Poland; ^3^Department of Molecular and Cellular Biology, Faculty of Biology, Institute of Molecular Biology and Biotechnology, Adam Mickiewicz University, Poznań, Poland; ^4^Laboratory of Ecology, Institute of Dendrology, Polish Academy of Science, Kórnik, Poland

**Keywords:** cell wall biogenesis, microarrays, Populus trichocarpa, wood, xylogenesis

## Abstract

Regulation of gene expression, as determined by the genetics of the tree species, is a major factor in determining wood quality. Therefore, the identification of genes that play a role in xylogenesis is extremely important for understanding the mechanisms shaping the plant phenotype. Efforts to develop new varieties characterized by higher yield and better wood quality will greatly benefit from recognizing and understanding the complex transcriptional network underlying wood development. The present study provides a detailed comparative description of the changes that occur in genes transcription and the biosynthesis of cell-wall-related compounds during xylogenesis in *Populus trichocarpa* pioneer roots and stems. Even though results of microarray analysis indicated that only approximately 10% of the differentially expressed genes were common to both organs, many fundamental mechanisms were similar; e.g. the pattern of expression of genes involved in the biosynthesis of cell wall proteins, polysaccharides, and lignins. Gas chromatography time-of-flight mass spectrometry (GC-TOF-MS) shows that the composition of monosaccharides was also very similar, with an increasing amount of xylose building secondary cell wall hemicellulose and pectins, especially in the stems. While hemicellulose degradation was typical for stems, possibly due to the intensive level of cell wall lignification. Notably, the main component of lignins in roots were guiacyl units, while syringyl units were dominant in stems, where fibers are especially needed for support. Our study is the first comprehensive analysis, at the structural and molecular level, of xylogenesis in under- and aboveground tree parts, and clearly reveals the great complexity of molecular mechanisms underlying cell wall formation and modification during xylogenesis in different plant organs.

## Introduction

*Populus* is an ecologically dominant and economically important tree species which also represents a perfect model for genomic studies of woody plants. Factors that make poplar an excellent model species include its modest genome size (∼500 M) (Taylor, 2002), ease of vegetative propagation, relative ease of transgenic manipulation, rapid growth, an extensive number of interspecific hybrids, and a large diversity of phenotypes (Hao et al., 2011). It is one of the fastest growing, temperate climate trees, and is capable of producing a high amount of biomass under poor climate and soil conditions (Hao et al., 2011). Wood (secondary xylem) is a dominant form of terrestrial biomass and is used in many industrial applications, ranging from pulp and paper production, to biofuels and biomaterials, and as a building material (Li et al., 2010; Plasencia et al., 2016).

Characteristics of each stage of xylogenesis have been defined at both a molecular and structural level (Fukuda, 2000; Oda and Hasezawa, 2006; Muñiz et al., 2008; Ohashi-Ito et al., 2010; Pesquet et al., 2013; Bagniewska-Zadworna et al., 2014; Serk et al., 2015; Wojciechowska et al., 2019). The majority of this information, however, has been obtained from plants grown in an artificial environment, such as *Zinnia* elegans (Fukuda, 2000; Kuriyama and Fukuda, 2002; Pesquet et al., 2013) and *Arabidopsis thaliana* (Ohashi-Ito et al., 2010) grown in *in vitro* culture where the process of tracheary element (TE) development was experimentally induced in cells, or from *A.* *thaliana* grown in a growth chamber or greenhouse (Bollhoner et al., 2013; Taylor-Teeples et al., 2015). Extending these data to the process of xylogenesis that occurs in plants grown in natural conditions is problematic and perhaps not reliable. While some studies of wood formation have been conducted in stems of poplar (Moreau et al., 2005; Courtois-Moreau et al., 2009; Tian et al., 2013; Wang et al., 2014) and eucalyptus (Carocha et al., 2015; Soler et al., 2016), knowledge is still lacking pertaining to xylogenesis in tree roots; especially in plants grown under field conditions.

The initiation and development of TEs is strictly regulated by genetically programmed processes that result in the formation of dead cells with thick secondary cell walls. Xylogenesis consists of different stages, including primary cell wall biosynthesis with cellulose and xylan deposition guided by microtubules (Oda et al., 2010; Pesquet et al., 2010), the expression of specific sets of genes associated with vascular development [e.g. TE differentiation-related (TED) family genes (Demura and Fukuda, 1993; Demura and Fukuda, 1994)], secondary cell wall formation, programmed cell death (PCD) resulting from the rupture of the tonoplast and the release of nucleases and proteases which degrade cytosolic structures (Fukuda, 1997; Fukuda, 2000; Obara et al., 2001; Ito and Fukuda, 2002; Bagniewska-Zadworna et al., 2012; Bagniewska-Zadworna et al., 2014; Wojciechowska et al., 2019), and lastly, *post mortem* lignification (Pesquet et al., 2013; Smith et al., 2013; Mishima et al., 2014). The process of xylogenesis is completed with the thinning and perforation of the end-wall of a TE, which is connected to the end wall of the next mature TE; thus forming a tubular xylem vessel that is able to conduct water and minerals (Esau and Charvat, 1978, Nakashima et al., 2000).

The primary cell wall (PCW) is the outermost layer of a plant cell and is extensively modified during cell growth. The PCW of a *Populus* xylem cell is an elastic, highly hydrated structure constructed mainly of carbohydrates, and is dominated by pectins such as homogalacturonan (HG) and rhamnogalacturonan I (RG-I). During the later stages of cell maturation, cellulose; an unbranched, linear β-1,4-linked glucan synthesized by cellulose synthase (CesA) complexes, and hemicelluloses; including xyloglucans, arabinoglucuronoxylan, and mannans, are synthesized and accumulate in PCW (Plomion et al., 2001; Caffall and Mohnen, 2009; Mellerowicz and Gorshkova, 2012; Schneider et al., 2016). Proteins are also present in the PCW and are divided into two classes: structural proteins (e.g. extensins and expansins) that are part of the cell-wall scaffolding; and enzymes that function in modifying the structure of the PCW (Caffall and Mohnen, 2009). The secondary cell wall (SCW) is constructed mainly of cellulose, and is enriched with hemicellulose, pectins, and lignins. After cellulose, lignin is the second most abundant plant biopolymer and is mainly present in the SCWs of specialized cells; including the TEs and fibers of vascular plants. Lignin formation occurs between cellulose microfibrils by the oxidation of lignin monomers that are synthesized *via* the phenylpropanoid pathway and secreted into the plant cell wall. Lignin is derived from three monomers, called monolignols: p-coumaryl alcohol, coniferyl alcohol, and sinapyl alcohol; which form H- (hydroxyphenyl), G- (guicyl), and S- (syringyl) units, respectively, in the lignin polymer. The lignin biosynthesis pathway has been thoroughly investigated and many proteins involved in the process of lignin deposition have been characterized. Lignin biosynthesis is initiated by L-phenylalanine ammonia-lyase (PAL) which catalyzes the conversion of phenylalanine to p-coumaryl CoA, and then continues *via* the action of cinnamate 4-hydroxylase (C4H) and 4-coumarate: coenzymeA ligase (4CL). This resulting intermediate product is then converted into p-coumaryl alcohol by caffeoyl-CoA O-methyltransferase (CCoaOMT), ferulate 5-hydroxylase (F5H), cinnamoyl-CoA reductase (CCR), and cinnamyl alcohol dehydrogenase (CAD) (Whetten and Sederoff 1995; Boerjan et al., 2003). Following their synthesis, monolignols are transported from the cytoplasm to the cell wall, where they are polymerized by peroxidases and laccases (Sterjiades et al., 1992; Sterjiades et al., 1993; Zhao et al., 2013).

For further practical applications, it is essential to understand how gene expression changes during the biosynthesis and differentiation of cell walls, which lead to the most visible and crucial stage of xylogenesis; the formation of the SCW. It is also crucial to determine exactly when and how all of the different polysaccharides and cell-wall-related proteins are deposited in the xylem cell walls of trees. Therefore, the aim of the present study was to create a comprehensive view of the mechanisms involved in cell wall biogenesis and modification in pioneer roots of *P. trichocarpa,* relative to these processes in stems. Pioneer roots in trees are long and thick (usually ≥ 1–2 mm diameter) first-order roots, which have been referred as “pioneer”, “long”, and “framework” roots (Lyford, 1980; Sutton and Tinus, 1983; Zadworny and Eissenstat, 2011). For these roots there are clearly defined stages of xylogenesis with daily rates of primary xylem TEs formation and determination of the moment of transition in secondary growth (Bagniewska-Zadworna et al., 2012; Bagniewska-Zadworna et al., 2014), that allows to perform comparative studies of xylogenesis process. Thus, in order to identify genes involved in xylogenesis, microarrays were used to perform transcript profiling. Moreover, the cell wall components during xylogenesis were quantified using gas chromatography time-of-flight mass spectrometry (GC-TOF-MS). Molecular probes for different cell wall components were also used to identify changes in cell wall structure and composition, as well as lignin autofluorescence to identify its presence and localization during primary and secondary growth of roots and stems.

## Results

### Functional Gene Groups Preferentially Expressed During Xylogenesis in Pioneer Roots

**Figure 1:**
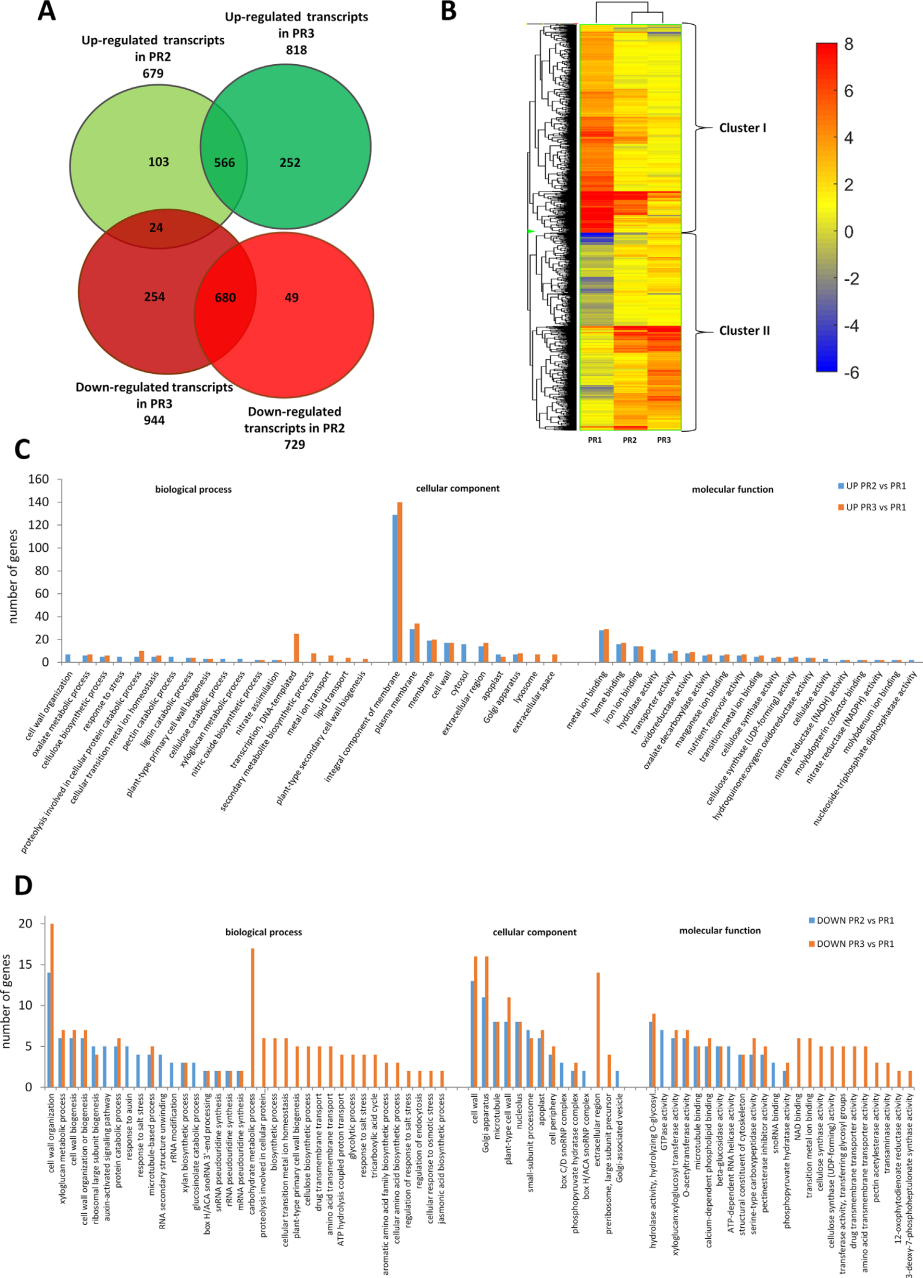
Global aspects of the black cottonwood transcriptome during three stages of xylem development in pioneer roots. **(A)** Expression pattern of the 1,914 statistically significant transcript sets at three time points during pioneer roots xylogenesis in black cottonwood and the overlaps among them are shown. Venn diagram comparing the differentially expressed transcripts. **(B)** Heatmap of 1914 *Populus trichocarpa* genes that showed, at last, two-fold differences with a p-value ≤ 0.001 in pioneer roots. Hierarchical clustering shows the gene expression clusters. **(C)** Functional classification of the genes up-regulated in the stage PR2 and PR3, respectively as compared to PR1 in pioneer roots of black cottonwood using the Database for Annotation, Visualization, and Integrated Discovery (DAVID). The x-axis represents the Gene Ontology (GO) annotation categories and the y-axis represents the number of total matched genes from a specific category. **(D)** Functional classification of the genes down-regulated in the stage PR2 and PR3, respectively as compared to PR1 in pioneer roots of *P. trichocarpa* using the DAVID. The x-axis represents the GO annotation categories and the y-axis represents the number of total matched genes from a specific category.

A poplar microarray was used to conduct a gene expression analysis of three pioneer root segments representing the different stages of xylogenesis to identify genes controlling xylem development. Among the 56,055 expressed genes that were detected; a total of 1,914 genes were differentially expressed during xylogenesis (one-way ANOVA, p ≤ 0.001, fold change ≥ 2). Among the differentially expressed genes (DEGs), 679 and 818 were up-regulated in the PR2 (primary growth of roots) and PR3 (secondary growth of roots) samples, respectively, relative to the control (PR1); while 729 were down-regulated in PR2 and 944 were down-regulated in PR3 samples (Figure 1A). A total of 1,386 of the DEGs were annotated and functionally classified using the Database for Annotation, Visualization, and Integrated Discovery (DAVID) (Huang et al., 2009a; Huang et al., 2009b). The analysis identified 88 functional clusters with a homogenous functional composition, including 78 Gene Ontology (GO) terms (**Supplemental Table S1**). The most abundant categories were those related to “in cell wall processes”, “proteolysis involved in cellular protein catabolic process,” and “carbohydrate metabolic process”. The majority of identified proteins were assigned to an integral component of the membrane, plasma membrane, and cell wall compartments.

Among the genes that were up-regulated, relative to the control (PR1), in the primary (PR2) and secondary (PR3) growths; 510 and 613 genes, respectively, could be annotated. Most of the genes that increased in expression in PR2 samples were also up-regulated in PR3 samples (**Figure 1A**). There was a significant group of genes, however, that were up-regulated only in PR2 or PR3. The most abundant GO categories were those related to cell wall biogenesis and development, and many of them were common to both the PR2 and PR3 stages of xylogenesis. These included “cellulose biosynthetic process” (CesA family protein, POPTR_0006s19580g and POPTR_0011s07040g; and CesA, POPTR_0002s25970g), “lignin catabolic process” (laccase 110b, POPTR_0006s08740g; laccase 3. POPTR_0010s20050g; a gene similar to At5g01050 encoding a laccase family protein, POPTR_0016s11520g; and a gene similar to At2g30210 encoding a laccase 3, POPTR_0019s14530g), and “plant-type PCW biogenesis” (CesA family protein, POPTR_0006s19580g; CesA, POPTR_0002s25970g and POPTR_0010s08550g). Important differences were also observed, however, in the expression of genes identified in the GO categories between the PR2 and PR3 stages. These included genes in GO categories such as “genes related to cell wall organization” (xyloglucan endotransglucosylase/hydrolase protein 31 precursor, POPTR_0009s01210g; and xyloglucan endotransglycosylase hydrolase 2 family protein, POPTR_0007s14570g), “pectin catabolic process” (a gene similar to At3g55140 encoding a pectate lyase family protein, POPTR_006s23090g; a gene similar to At3g59010 encoding a pectinesterase family protein, POPTR_0014s14720g), and “xyloglucan metabolic process” (a gene similar to At4g02290 encoding glycosyl hydrolase 9B13, POPTR_0001s11430g); all of which were up-regulated only in the PR2 samples. In contrast, other genes such as those involved in “regulation of transcription” (ERF domain protein 12, POPTR_0004s04700g; WOODY family protein, POPTR_0008s08130g; a gene similar to At5g18240 encoding MYB-related protein 1; NAC domain proteins, POPTR_0001s45250g, POPTR_0002s15530g, and POPTR_0004s049001g; a gene similar to At2g33860 encoding ETTIN, POPTR_0011s05830g; a gene similar to At4g18890 encoding brassinosteroid signaling positive regulator-related gene, POPTR_0011s06800g; a gene similar to At2g01060 encoding a myb family transcription factor, POPTR_0016s12430g; a gene similar to At2g38560 encoding TRANSCRIPT ELONGATION FACTOR IIS, POPTR_0016s14510g; a myb family transcription factor family protein gene, POPTR_0010s20040g, and a no apical meristem family protein gene, POPTR_0007s01350g), “secondary metabolite biosynthesis” (cytochrome genes P450, POPTR_0001s16790g and POPTR_0002s02790g), and “plant-type SCW biosynthesis” (a gene similar to At3g18660 encoding PLANT GLYCOGENIN-LIKE STARCH INITIATION PROTEIN 1, POPTR_0005s06280g; and CesA PtiCesA3-1, POPTR_0011s07040s) were only differentially expressed in PR3 samples. The results described above indicated the diversity of gene expression involved in primary and SCW biogenesis (**Figure 1C**). Additionally, genes related to “proteolysis involved in cellular protein catabolism” (cysteine endopeptidase family protein, POPTR_0015s09890g; serine carboxypeptidase precursor family protein, POPTR_0008s03480g; a gene similar to At4g16190 encoding a cysteine proteinase, POPTR_0005s25540g; a gene similar to At5g08260 encoding serine carboxypeptidase-like 35, POPTR_0007s07690g; and a gene similar to At5g50260 encoding a cysteine proteinase, POPTR_0015s09900g) increased in expression during both the primary and secondary growths; suggesting that PCD is an essential part of xylogenesis during wood development (**Figure 1C**).

The majority of the 729 and 944 genes down-regulated in PR2 and PR3 were down-regulated in both stages (**Figure 1A**). Among them, however, were genes involved in cell wall biogenesis and development; indicating the existence of a variety of mechanisms underlying primary and SCW development. Among the down-regulated genes were those that were only down-regulated in PR3 samples (**Figure 1D**), including those that were involved in “xyloglucan and cellulose biosynthetic processes” (xyloglucan endotransglycosylase hydrolase 2 family protein, POPTR_0007s14570g; PtiCesA7A-like family protein, POPTR_0005s21620g; CesA 6 family protein, POPTR_0013s02050g; CesA family protein, POPTR_0001s37770g; and CesA family protein, POPTR_0007s07120g). We also identified genes involved in the early stage of PCD (aspartyl protease family protein, POPTR_0003s07390g and POPTR_0004s08340g; a gene similar to At5g45120 encoding an aspartyl protease family protein, POPTR_0012s11860g and POPTR_0015s12480g; a gene similar to At2g27920 encoding serine carboxypeptidase-like 51, POPTR_0009s00820g; a gene similar to At3g63470 encoding serine carboxypeptidase-like 40, POPTR_0009s06080g; and serine carboxypeptidase S10 family protein, POPTR_0002s07260g and POPTR_0010s22680g). Some of those involved in protein catabolic processes were already down-regulated in samples representing the primary and the secondary growths (including primary or secondary xylem); but others associated with the proteolysis involved in the cellular protein catabolic process that occurs during the later phase of PCD were only down-regulated in PR3 stage (aspartyl protease family protein, POPTR_0010s13830g; a gene similar to At4g30810 encoding serine carboxypeptidase-like 29, POPTR_0018s11210g; and serine carboxypeptidase S10 family protein, POPTR_0008s12860g). GO analysis indicated that the identified genes function in the cell wall and plant-type cell wall, Golgi apparatus, microtubule, and nucleolus categories of the larger “cell component” category. A total of 14 Kyoto Encyclopedia of Genes and Genomes pathways were identified, some of which were identified in both PR2 and PR3 samples; suggesting decreased metabolic activity during the course of xylem maturation. The down-regulated genes were associated with categories such as “biosynthesis of secondary metabolites” (chain A family protein, POPTR_0015s00550g; cytochrome P450 family protein, POPTR_0001s20720g; cytokinesis defective 1 family protein, POPTR_0008s06050g; and gibberellin 3 beta-hydroxylase family protein, POPTR_0006s26380g), “biosynthesis of amino acids” (2-dehydro-3-deoxyphosphoheptonate aldolase family protein, POPTR_0005s17270g; 3-phosphoshikimate 1-carboxyvinyltransferase family protein, POPTR_0002s14740g; and citrate synthase family protein, POPTR_0001s23760g), “carbon metabolism” (NAD-dependent malic enzyme family protein, POPTR_0014s07570g; succinate dehydrogenase flavoprotein subunit, POPTR_0007s12750g; and alcohol dehydrogenase family protein, POPTR_0002s01480g), “ribosome biogenesis in eukaryotes” (dyskerin family protein, POPTR_0006s04270g and POPTR_0016s04070g; and a gene similar to At5g37350 encoding a Rio1 family protein POPTR_0015s10810g), and “phagosomes” (ATP synthase subunit H family protein, POPTR_0011s09510g; vacuolar ATP synthase subunit G 2 family protein, POPTR_0010s22880g; vacuolar ATP synthase subunit B family protein, POPTR_0004s18400g; vacuolar ATPase subunit E family protein, POPTR_0019s04470g; and vacuolar proton ATPase family protein, POPTR_0009s12430g). Genes related to the phenylpropanoid pathway (peroxidase 27 precursor family protein, POPTR_0019s09150g; peroxidase precursor family protein, POPTR_0002s06590g; and a gene similar to At3g01190 encoding peroxidase 27, POPTR_0004s02240g) were down-regulated in PR3 samples; indicating that the expression of some genes involved in lignin biosynthesis had already decreased.

### Functional Gene Groups Preferentially Expressed During Xylogenesis in Stems

**Figure 2:**
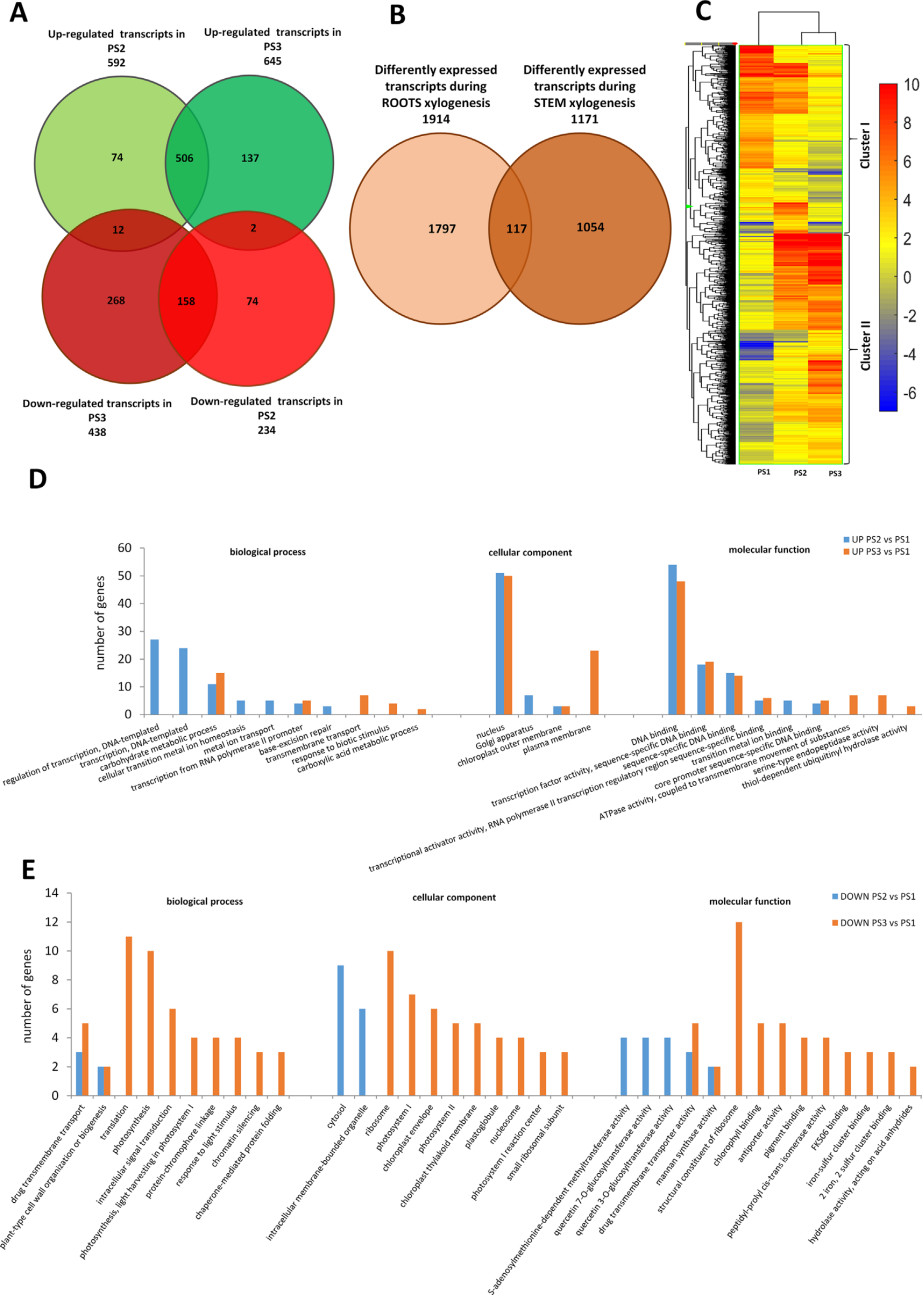
Global aspects of the black cottonwood transcriptome during three stages of xylem development in stem. **(A)** Expression pattern of the 1171 statistically significant transcript sets at three time points during pioneer roots xylogenesis in black cottonwood and the overlaps among them are shown. Venn diagram comparing the differentially expressed transcripts. **(B)** Expression pattern of statistically significant transcript sets in pioneer roots and stem of black cottonwood during xylogenesis process and the overlaps among them are shown. Venn diagram comparing the differentially expressed transcripts. **(C)** Heatmap of 1,171 *Populus trichocarpa* genes that showed, at last, two-fold differences with a p-value ≤ 0.001 in stem. Hierarchical clustering shows the gene expression clusters. **(D)** Functional classification of the genes up-regulated in the stage PR2 and PR3, respectively as compared to PR1 in pioneer roots of black cottonwood using the Database for Annotation, Visualization, and Integrated Discovery (DAVID). The x-axis represents the GO annotation categories and the y-axis represents the number of total matched genes from a specific category. **(E)** Functional classification of the genes down-regulated in the stage PR2 and PR3, respectively as compared to PR1 in pioneer roots of *P. trichocarpa* using the DAVID. The x-axis represents the GO annotation categories and the y-axis represents the number of total matched genes from a specific category.

Transcripts expressed in three shoot segments representing the different stages of xylogenesis were analyzed to obtain an overall view of DEGs during xylem formation in poplar stem. In total, 1,171 genes were differentially regulated in PS2 and PS3 stages, relative to PS1 stage, in the examined samples (one-way ANOVA, p ≤ 0.001, fold change ≥ 2) among the 56,055 transcripts present on the poplar microarray. Among the DEGs, 592 were up-regulated in the PS2 stage (secondary growth) and 645 in the PS3 stage (isolated secondary xylem), while 234 were down-regulated in PS2 and 438 in PS3 stage (**Figure 2A**). A total of 896 DEGs could be annotated, and 55 clusters and 42 functional categories were identified for these genes using DAVID v6.8 analysis software (Huang et al., 2009a; Huang et al., 2009b) (**Supplemental Table S2**). GO analysis was performed to investigate whether the DEGs in the PS2 and PS3 samples are involved in similar processes. The GO enrichment analysis revealed that the most abundant categories were those related to “DNA-templated regulation of transcription” (GO: 0006355), “carbohydrate metabolic process” (GO: 0005975), and “flavonoid metabolic process” (GO: 0052696 and GO: 0009813).

In stems, significantly more genes were up- than down-regulated during xylogenesis; whereas an opposite tendency was observed in pioneer roots (**Figure 2A** *vs.* **Figure 1A**). Most transcripts up-regulated in PS2 samples were also significantly up-regulated in PS3 samples (**Figure 2A**), although there was a significant group of genes that were up-regulated only in PS3 samples. The most abundant GO categories identified in PS2 samples were those related to transcription and metal ions (**Figure 2D**). A common functional class represented in both PS2 and PS3 samples was carbohydrate metabolic process. Most processes up-regulated during stem xylogenesis occur in the plasma membrane and nucleus. There were two GO BP categories that were down-regulated in both PS2 and PS3 samples: “drug transmembrane transport (GO: 0006855),” and “plant-type cell wall organization or biogenesis (GO: 0071669)” (**Figure 2E**). The process of xylem development appeared to be more differentiated in PS2 *vs.* PS3 samples. Most of the transcripts that were down-regulated in the PS2 stage of xylogenesis were also down-regulated in PS3 (**Figure 2A**); whereas, most of the genes that decreased in isolated wood (PS3) were specific to that stage. The most abundant common GO categories in PS2 and PS3 samples were those related to “drug transmembrane transport” [multi-antimicrobial extrusion protein (MATE) efflux family protein, POPTR_0013s06650g; and genes similar to At3g03620 encoding a MATE efflux family protein, POPTR_0013s06540ga and POPTR_0013s06560g] and “plant-type cell wall organization or biogenesis” (CesA family proteins, POPTR_0002s20130g and POPTR_0014s12000g) (**Figure 2E**). The functional classes of genes specific to PS3 were mostly those associated with translation, which together with the fact that “ribosome” was the most abundant GO CC category, suggest that protein biosynthesis was ceasing as PCD was being initiated in xylem cells. Other important GO categories associated with genes down-regulated in PS3 were those related to photosynthesis and chloroplasts; suggesting the shutdown of photosynthetic activity in mature xylem cells.

### Expression Profile of Cell Wall-Related Genes in Pioneer Roots and Stems

Clusters of co-regulated genes were identified by hierarchical clustering analysis of 1,914 and 1,171 DEGs from roots and stems, respectively, to obtain an overview of the expression data set. Hierarchical clustering, based on expression response across conditions, revealed a clear separation between genes expression in stems *vs.* roots. Cluster I in roots is composed of 982 genes that were mostly down-regulated in PR2 and PR3 samples relative to PR1 samples. Cluster II in roots is composed of 932 mostly up-regulated genes (**Figure 1B**). Similarly, in stem samples, cluster I is composed of 526 genes down-regulated in PS2 and PS3 relative to their expression in PS1 samples; while cluster II contains 645 up-regulated genes (**Figure 2C**). The clusters were analyzed for over-representation of functional GO categories using the PANTHER database (Mi et al., 2016). Results indicated a significant over-representation of genes related to cell wall organization and biogenesis in both clusters in pioneer root samples but not in stem samples; where the most abundant GO categories were related to regulation of transcription (**Supplemental Tables S3** and **S4**). Based on the results of the functional analyses described in the paragraphs above, and the analysis of over-representation of GO terms, a closer examination of genes involved in cell wall metabolism in pioneer roots and stem samples was conducted.

#### Genes Associated With Cell Wall Protein Metabolism

Genes related to cell wall structural proteins were identified in cluster I of root and stem samples that were down-regulated in PR2 and PR3 samples. Only a few genes (*alpha-expansin*, *beta expansin*, *AGP9*, *AGP10*, *PtrFLA1,* and *PtrFLA24*) were up-regulated and assigned to cluster II, suggesting that they are mostly involved in the primary growth. Hydroxyproline-rich glycoproteins (HRGPs) have been identified as important structural components of cell walls. The expression of six and seven genes encoding HPRGs were identified as being down-regulated in PR2 and PR3 (cluster I) root samples, respectively, and a single gene that was up-regulated (cluster II) was identified in PR2 and PR3 samples; suggesting that they may play a role in PCW development. In contrast, the expression of only two HPRG genes was altered in stem samples; one gene (POPTR_0014s12960g) decreased (cluster I) and one (POPTR_0010s00550g) increased (cluster II) in expression in both PS2 and PS3 samples. Extensins (HRGPs) are also a group of proteins of great importance to cell wall architecture and constitute the major protein component of cell walls of dicots. The expression of extensins is tissue-specific and/or development-specific, and affected by various conditions and treatments. In the current study, a significant down-regulation of extensin-coding genes involved in root hair morphogenesis, elongation, and cell wall organization was observed in cluster I of both root and stem samples. The expression of extensin genes in roots, however, was greater than it was in stems. Expansins are another group of cell-wall structural proteins that play an important role in plant cell growth and developmental processes that involve cell wall loosening, such as cell wall modification during xylogenesis. Genes encoding expansin S1 precursor, as well as an expansin-related protein 1 precursor, were down-regulated in PR2 and PR3 samples of roots and grouped together in cluster I. On the other hand, genes encoding an expansin-like B2 precursor, beta-expansin precursor, and three alpha-expansin genes were up-regulated in both PR2 and PR3 (cluster II) root samples. Genes encoding expansin and an alpha-expansin precursor were down-regulated (cluster I) in PS2 and PS3 stem samples and only an expansin-like B1 gene was up-regulated (cluster II) in PS3 samples. Collectively, the results suggest that expansins may play an important role in cell wall modification during xylogenesis in roots but not in stems.

Arabinogalactan proteins are a highly diverse class of cell surface proteoglycans commonly found in the PCWs of most plant species. Most genes encoding arabinogalactan proteins (AGPs) in roots were down-regulated and grouped in cluster I. However, two *AGP*s, *AGP*9 and *AGP10,* were identified that were up-regulated (cluster II) in PR2 and PR3 root samples. Since the expression level of these genes was rather low, it is plausible that they primarily play a role in the early stages of xylogenesis in roots. Genes encoding AGP 9 and 10 were up-regulated (cluster II) in stem samples; suggesting that they may play a role in the later stages of cell wall development. Fasciclin-like AGPs (FLAs) are located in the cell wall and are involved in many developmental processes, however, their exact role has not been fully confirmed. Some FLAs, however, have been linked to SCW development. In the current study, seven *FLA* genes were identified in root samples; four were down-regulated and grouped in cluster I, and three exhibited increased expression and were accordingly assigned to cluster II. Taken together, these observations suggest that they are involved in SCW development during both the primary and secondary growth. In contrast, 17 *FLAs* were identified in stem samples, and all but one were up-regulated (cluster II); also indicating their role in secondary wood development.

#### Genes Involved in Cell Wall-Related Carbohydrate Metabolism

##### Cellulose

**Figure 3:**
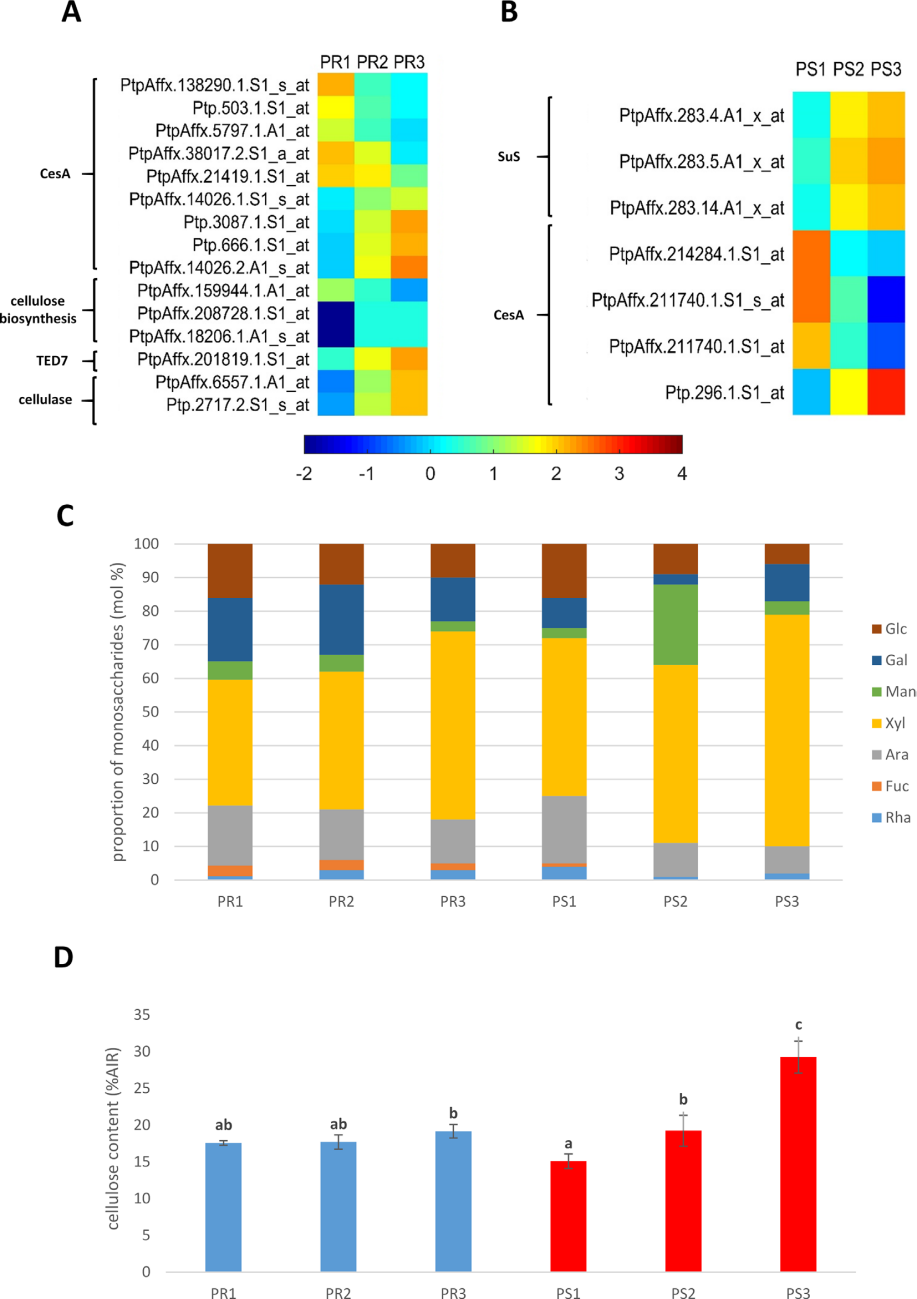
**(A)** Expression profile of genes related to cellulose metabolism in pioneer roots and **(B)** stem of black cottonwood. CesA, cellulose synthase; TED7, tracheary element differentiation-related 7 membrane protein; SuS, sucrose synthase. **(C)** Cell wall monosaccharide composition in pioneer roots and stem of *Populus trichocarpa*. Cell wall monosaccharide composition was measured from alcohol-insoluble residues extracted from root and stem tissues. Rha, rhamnose; Fuc, fucose; Ara, arabinose; Xyl, xylose; Man, mannose; Gal, galactose; Glu, glucose. **(D)** Cellulose content in cell walls in pioneer roots and stem of *P. trichocarpa*. Crystalline cellulose content was measured in alcohol-insoluble residue (AIR) of cell walls with Updegraff method. Means designated by different letters indicate changes statistically significant according to Tukey´s *post hoc* test, (P < 0.05). Error bars indicate ± SE (n = 4).

***Roots.*** Significant differential expression of genes encoding CesA were observed in pioneer roots. Ten genes were down-regulated (cluster I), which suggests that they have a role in primary growth, with primary xylem differentiation. Among the 10 genes that were identified, two encoded *Ces7a-like* genes which are usually associated with SCW formation. An additional four *CesA* genes were up-regulated (cluster II), including *PtiCesA3-1*, which is associated with PCW formation; however, it was up-regulated in PR3 samples. Additionally, expression of the gene encoding the TE Differentiation-Related7 TED7 membrane protein, which is associated with a *CesA* complex in SCW formation, was also up-regulated (cluster II) in roots. Two cellulase-coding genes were also grouped in cluster II. The observed pattern of gene expression indicates a high level of cellulose biosynthesis during primary and secondary growth, as well as cellulose decomposition required during cell wall remodelling during the course of xylem lignification and maturation (**Figure 3A**).

***Stems. ***Differential expression of sucrose synthase genes, which provide uridine diphosphate (UDP)-glucose substrate for CesA, was observed in stem samples. Expression of three genes similar to *Arabidopsis Sucrose Synthase1* was also identified. These genes were up-regulated (cluster II) in PS2 and PS3 samples; suggesting their involvement in both primary and secondary growth of stems. A lower number of *CesA* genes were identified in stem samples* vs.* roots. Four were identified and three of them were down-regulated (cluster I) in PS2 and PS3 samples; indicating that they are likely involved in PCW biosynthesis. In contrast, one of the *CesA* genes was up-regulated (cluster II) in both PS2 and PS3 samples; indicating its role in both primary and secondary growth of stems (**Figure 3B**).

##### Hemicellulose

Hemicellulose synthesis-related genes were expressed in both roots and stems but their expression pattern was significantly different in the two organs. They were down-regulated (cluster I) in PR2 and PR3 root samples (cluster I) but up-regulated (cluster II) in PS3 stem samples. The expression of nine genes encoding β-glucosidase, which play a role in hemicellulose degradation, were also found to be down-regulated (cluster I) in pioneer roots; suggesting that hemicellulose biosynthesis and metabolism play a significant role in primary xylogenesis in pioneer roots. Xylan, a type of hemicellulose, is another important component of primary and SCWs. Five xylan biosynthesis-related genes were identified in pioneer roots, three of which were down-regulated (cluster I), including IRREGULAR *XYLEM 14* (*IRX14*), and three that were up-regulated (cluster II), including *IRX9.* These observations suggest that xylan is synthesized during development of primary and secondary xylem. *Glucuronic acid substitution of xylan 2* (*GASX2*—plant glycogenin-like starch initiation protein 3 PGLSIP3), associated with xylan biosynthesis, was identified in root samples and its expression was down-regulated (cluster I) in PR2 and PR3 samples, while the expression of *PGLSIP* was up-regulated (cluster II) in PR3 samples. A candidate transcription factor associated with xylan biosynthesis, similar to *Arabidopsis WRKY TF35*, was also identified in cluster II in roots. Collectively, these data suggest that they play a role in both primary and SCW formation in pioneer roots. The expression of the xylan biosynthesis-related gene and *PGLSIP1* were both up-regulated (cluster II) in PS2 and PS3 stem samples. A gene encoding xylanase, however, was also up-regulated (cluster II) in PS2 and PS3 stem samples; indicating that xylan and hemicellulose are broken down during cell wall remodelling. It appears that xylan biosynthesis may be more active during primary xylogenesis in pioneer roots than in stems. Xyloglucan is another essential constituent of the PCW. Expression of a gene encoding an endoxyloglucan transferase involved in xyloglucan biosynthesis was down-regulated (cluster I) in PR2 and PR3 root samples; suggesting that its activity is important during early cell wall biosynthesis. Genes encoding xyloglucan endo-transglucosylase are involved in the cleavage and regulation of xyloglucan polymers. They were up-regulated in PR2 and down-regulated in the latter stage of cell wall development (PR3); suggesting that cell wall remodelling due to xyloglucan degradation occurs only during the primary growth.

##### Pectins

Three β-D-xylosidase family proteins, involved in pectic arabinan modification during vascular development, were down-regulated (cluster I) in PR2 and PR3 stage root samples. However, two other β-xylosidase genes were highly up-regulated (cluster II) in both stages; suggesting that they play a role in cell wall remodelling during both the primary and secondary growth of pioneer roots. The significance of the genes in this category has not been demonstrated in stems. Nine genes involved in HG metabolism were identified in pioneer rots in the current study. A glycosyltransferase belonging to the CAZy family and implicated in the synthesis of pectins and xylan was found to be down-regulated in PR2 and PR3 stages (cluster I). Two glycosyltransferase 2 family protein genes involved in HG biosynthesis were also found to be down-regulated in PR2 and PR3 samples. Three other genes encoding glycosyltransferase 2 family proteins were down-regulated only in PR3 samples (cluster I). A UDP-glucuronic acid/UDP-N-acetylgalactosamine transporter-related gene was also down-regulated in PR3 stage (cluster I); whereas, *glycosyltransferase 37* was significantly induced in PR2 and PR3 samples (cluster II). While in stem samples, four genes involved in HG biosynthesis were identified. The expression of one UDP-glycosyltransferase gene was down-regulated in PS2 and PS3 samples (cluster I); however, the other one was up-regulated in PS2 stage and grouped together in cluster II with three glycosyltransferase family 8 protein genes that were up-regulated in PS2 and PS3 samples. The expression of a gene encoding galactan synthase 1 was down regulated (cluster I) in PR2 and PR3 samples; suggesting that this stage of pectin biosynthesis may already be completed prior to the secondary xylem formation in pioneer roots.

Pectinesterase is a cell wall-related enzyme that facilitates cell wall modifications by catalyzing the de-esterification of pectin. The expression of two genes encoding a pectinesterase precursor and five genes encoding pectinesterase was down-regulated in PR2 and PR3 root samples (cluster I). These data suggest that those genes are no longer involved in the later stages of xylogenesis or that pectinesterase precursors are no longer needed in the later stages of xylogenesis; hence their decreased level of expression. Three pectinesterase genes, however, were up-regulated only in PR2 samples; indicating that they only play a role during the primary growth. One pectinesterase gene was also up-regulated in PR3 stage (cluster II) which suggests that it may play a role in secondary xylem development. Changes in the expression of genes encoding pectinesterases were not observed in stem samples. Pectin methylesterases (PMEs) are another group of enzymes that are active during the biosynthesis and modifications of cell walls. PMEs are responsible for the de-methylation of pectin resulting in a relaxation of the cell wall which is needed for cells to enlarge. All five genes encoding PME were up-regulated in PR2 and PR3 root samples (cluster II), suggesting that an intensive modification of the cell wall occurs at that time. Genes encoding a PME inhibitor were also identified in roots, one of which was down-regulated in PR2 and PR3 (cluster I) and two that were up-regulated (cluster II) in PR2 or PR3 stage; consequently inhibiting PME in PR2 and PR3 samples, respectively. Only one PME gene with down-regulated (cluster I) expression was identified in stem samples in all the stages of xylogenesis, while two genes encoding PME inhibitors exhibited increased expression (cluster II); thus indicating that PME plays a role in SCW modifications but does not play a significant role in mature xylem (PS3 stage) in stems. Pectinacetylesterase is a cell-wall-associated enzyme that is responsible for the deacetylation of pectin. This modification increases the mechanical strength of cell walls by decreasing pectin-related elasticity. All but one gene encoding a pectin acetylesterase were down-regulated (cluster I) in PR2 and PR3 root samples, suggesting that a high level of pectin acetylation and increased cell wall elasticity is needed during xylogenesis. Changes in the expression of pectin acetylesterase genes were not observed in stem samples. Pectinase is a cell-wall-degrading enzyme that breaks down pectins. Genes encoding pectinase were up-regulated in root samples (cluster II), while a pectate-lyase like and pectin catabolic related genes were down-regulated (cluster I). Similarly, two genes encoding a pectinase were up-regulated (cluster II) in stem samples; suggesting that they play a role in the cell wall relaxation required for cell wall modifications to occur during xylogenesis.

#### Genes Involved in Lignin Metabolism

**Figure 4:**
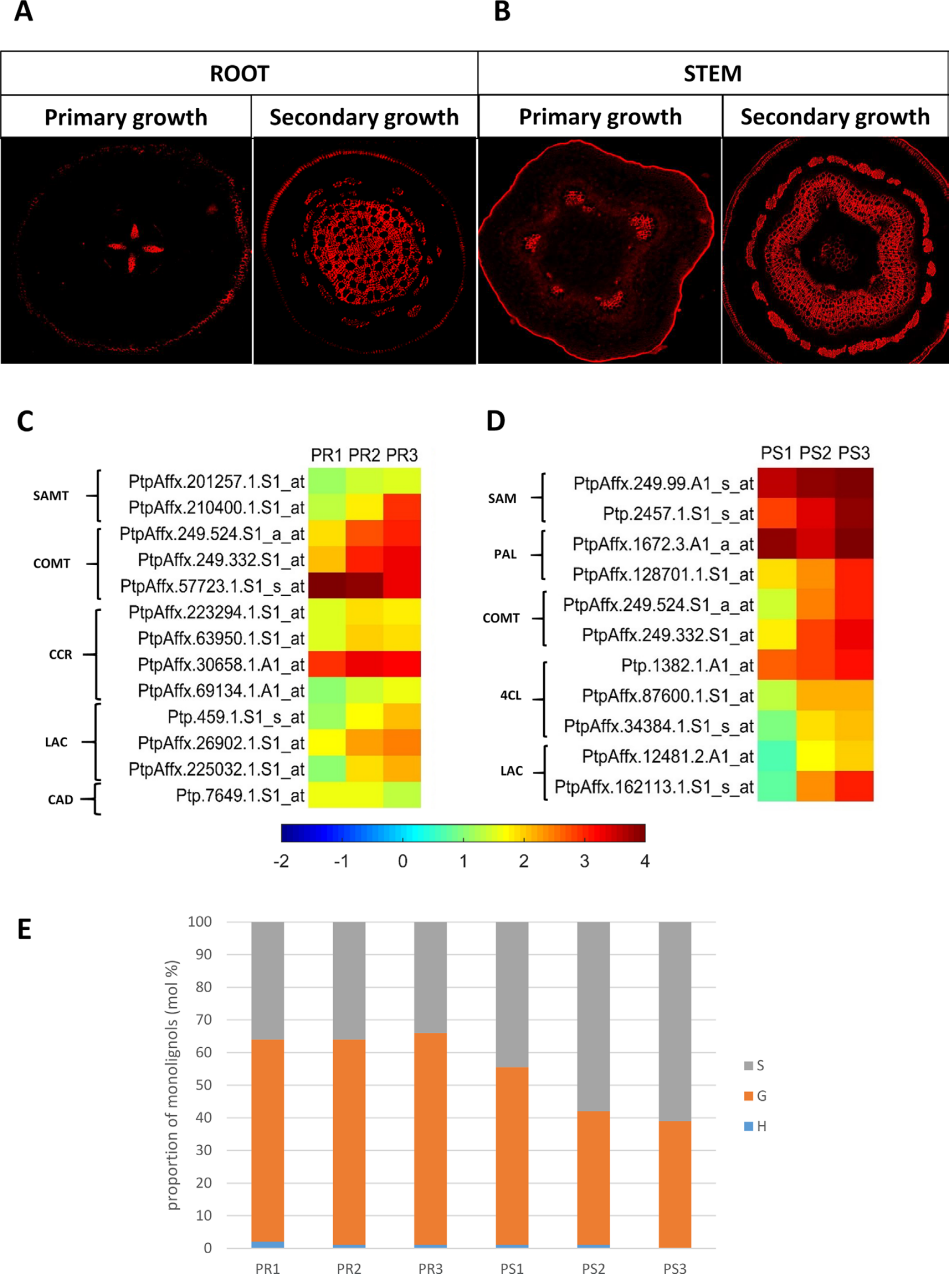
**(A)** Autofluorescence of lignins in pioneer roots and **(B)** stem of *Populus trichocarpa*. **(C)** Expression profile of genes related to lignin metabolism in pioneer roots and **(D)** stem of black cottonwood. SAMT, S-adenosyl-L-methionine:carboxyl methyltransferase; COMT, caffeic acid O-methyltransferase; CCR, cinnamoyl-CoA reductase; LAC, laccase; CAD, cinnamyl alcohol dehydrogenase; SAM, S-adenosylmethionine synthetase; PAL, phenylalanine ammonia-lyase; 4CL, 4-coumarate:CoA ligase. **(E)** Cell wall lignin composition in pioneer roots and stem of *P. trichocarpa*. Cell wall lignin composition was measured from alcohol-insoluble residues extracted from stem and root tissues. H (p-hydroxyphenyl), G (guaiacyl), S (syringyl) lignin monolignols.

In both pioneer roots and stems, the level of lignin autofluorescence increased along with the development of the secondary structures (**Figures 4A, B**). Genes in roots that were up-regulated in PR2 and PR3 root samples (cluster II), relative to the PR1 control samples, were subjected to a GO analysis. The analysis identified genes that are enriched in the category SCW metabolism, and in particular, lignin biosynthesis. *S-adenosyl-L-methionine:carboxyl methyltransferase, caffeic acid O-methyltransferase catechol-O-methyltransferase (COMT), CCR,* and *laccases* were all present in this cluster. Cluster I, which also possessed genes related to lignin biosynthesis, included a gene encoding COMT and one CAD-like protein whose expression decreased in PR3 samples (**Figure 4C**). Very similar results were obtained for stem samples, where genes encoding S-adenosylmethionine synthetase 2, PAL, COMT, 4-coumarate:CoA ligase 4CL, and laccase were up-regulated in PS2 and PS3 samples and grouped together in cluster II. Only one gene encoding PAL was down-regulated in PS2 and grouped in cluster I (**Figure 4D**). Even though the majority of genes related to lignin biosynthesis were up-regulated (cluster II) during the primary and secondary growth, a greater increase in their expression was observed during secondary xylem formation in both organs.

#### Transcription Factors Involved in Xylogenesis

The expression of transcription factors that may regulate the spatial distribution of pathways involved in xylogenesis was examined in order to identify the potential “master” genes involved in this process. NAC and MYB transcription factors have been reported as master switches in downstream pathways of SCW formation.

Eight genes encoding NAC domain proteins in roots were up-regulated (cluster II), while one was down-regulated (cluster I); suggesting that these transcription factors play an important role in xylogenesis in roots. A single *NAC* transcription factor was observed in stems that was down-regulated in PS2 and PS3 samples, while four NAC domain proteins were up-regulated only in PS2 samples. Two NAC proteins with significant similarity to *Arabidopsis NAC10* and *NAC97* were up-regulated in PS2 and PS3 stem samples.

Numerous MYB transcription factors were also identified in roots that were up-regulated in PR2 and/or PR3 samples (17 *MYB*s in total), while 15 *MYB*s were down-regulated. Among the *MYB* genes, *PtrMYB088*, *PtrMYB109*, and *PtrMYB122* highly similar to *AtMYB36/92/70*, respectively, as well as putative *MYB* transcription factors with high sequence identity to *Arabidopsis MYB19/109/122* and *MYBL2* were identified that were down-regulated in root samples. Other putative candidate genes corresponding to *PtrMYB010/084/133/139/152* transcription factors that are similar to *AtMYB103/82/84/40/43* and *MYR1* protein, and that were up-regulated in PR2 and/or PR3 samples, were also identified in roots. For example, a putative *PtrMYB184* that is similar to *AtMYB38,* which is known to regulate axillary meristem formation, was identified in the pioneer roots. In addition, *altered phloem development (APL)* was also found to be up-regulated in PR2 samples. *APL* is *MYB*-type TF that functions as a key regulator of phloem identity, moreover as suggested by Bonke et al. (2003), it also acts as a repressor of xylem differentiation while promoting phloem formation. This suggests that the process of xylem differentiation is inhibited at the PR2 stage of xylogenesis. Importantly, 29 *MYB* transcription factors were differentially expressed in stems: 4 were down-regulated (including *PtrMYB038* and *PtrMYB083* similar to *AtMYB106* and *AtMYB16*, respectively) and 25 were up-regulated; possibly suggesting the increased expression of genes involved in downstream pathways regulating SCW formation. This may result in the more abundant development of secondary xylem in stems relative to roots. *PtrMYB128* (similar to *AtMYB26*), involved in the formation of secondary wall thickenings and *MYB85* which controls secondary cell-wall formation, were also identified in stems. An *APETALA2/ethylene response factor AP2/ERF* transcription factor involved in the regulation of plant growth and development was identified in root and stem samples. In pioneer roots, 10 genes encoding *AP2/ERF* TFs were identified. Most of them grouped in cluster I and were down-regulated in both PR2 and PR3 samples, while three *AP2/ERF* genes were up-regulated during both the primary and secondary growth; suggesting their greater role at the beginning of the process of xylogenesis. In contrast, six *AP2/ERF* genes were found in stems; three of which were down-regulated and grouped in cluster I, and three others that were up-regulated and grouped in cluster II. These findings suggest that *AP2/ERF* is involved in both stages of xylogenesis in stem and are actively transcribed even in mature xylem (PS3 stage); which also indicates their potential involvement in *post mortem* lignification. *WRKY* transcription factors are important transcriptional regulators in plants and key players of signaling networks that regulate many plant processes. WRKY proteins may act both as repressors and activators. Most WRKY genes *(WRKY 18*, *23,* and *45*) in pioneer roots were up-regulated and grouped in cluster II, while the expression of *WRKY 35*, *48,* and *59* was down-regulated and grouped in luster I. All of the *WRKY* genes identified in stem samples were up-regulated, with significant up-regulation of *WRKY 17* and *75* in mature xylem (PS3); suggesting their role in the late phase of xylogenesis. Six zinc finger C2H2 type genes were identified in pioneer roots, all but one of which were up-regulated in both PR2 and PR3 stages; suggesting their role during both the early and late stages of wood development. Only three of these types of genes were found in stem samples, two of which were up-regulated in both PS2 and PS3 samples, and one that was up-regulated in PS2 samples but down-regulated in PS3 samples.

Another group of TFs that were observed to be expressed during xylogenesis were basic/helix-loop-helix (bHLH) proteins. Fifteen *bHLH* genes were identified in pioneer roots, eight that were down-regulated (including poplar orthologs of *MUTE*, *BIGPETAL,* and *PHYTOCHROME-INTERACTING FACTOR7*) and seven that were up-regulated (e.g. poplar orthologs of *CRYPTOCHROME-INTERACTING BASIC-HELIX-LOOP-HELIX 1* and *EMBRYO DEFECTIVE 1444*). A slightly fewer number of *bHLH* genes were found in stems but their expression patterns were more distinct. Two were only down-regulated in PS3 samples (including poplar homolog of *unfertilized embryo sac 10*), one was down-regulated in PS2 samples (gene similar to At1g68920), two were up-regulated only in PS2 samples, and four others were down-regulated during the whole process of xylogenesis (poplar orthologs of abscisic acid (*ABA*)*-INDUCIBLE BHLH-TYPE TRANSCRIPTION FACTOR* and ALCATRAZ). These data suggest that *bHLH* TFs play a role in many different processes throughout the entire process of xylogenesis. HD-ZIP III transcription factors play an essential role in many different developmental processes, including xylem formation. *PHABULOSA (PHB*) belongs to the HD-ZIP III class of TFs and is a positive regulator of cell wall secondary thickening and therefore secondary xylem development. Expression of *PHB* was up-regulated in pioneer roots. SHORT ROOT *SHR* was down-regulated in roots and *SCARECROW SCR,* belonging to the GRAS transcription factor family controlling the determination of meta- and protoxylem by regulating *HD-ZIP III* expression, was up-regulated in roots. *KANADI* belonging to the GARP family (a subgroup of MYB) is expressed in phloem, and by mutual negative interaction with HD-ZIP IIIs, participates in regulating the adaxial/abaxial orientation of phloem and xylem. Expression of this gene was not down-regulated in stems until the late stage of xylem development (PS3).

#### Genes Related to Cell Wall Development That Were Common to Pioneer Roots and Stems

A comparison of the GO categories between pioneer roots and stem indicated that all but one (carbohydrate metabolic process GO: 0005975) were specific to either roots or stems. Therefore, the lists of DEGs identified during xylogenesis in pioneer roots and stems were compared. The analysis revealed that out of 1,914 DEGs in roots and 1,171 DEGs in stem, only 117 were common to both organs (**Figure 2B**). Among those genes in common were those related to cell wall biogenesis and development, such as genes encoding COMT that are involved in the lignin biosynthesis pathway, transcription factors considered to be regulators of primary and SCW development, including *PtrFLA24* (Zang et al., 2015) and *PtrMYB167* (Chai et al., 2014), a leucine-rich repeat transmembrane protein kinase linked to SCW biogenesis, a no apical meristem family protein involved in the regulation of SCW biogenesis, and a protein similar to trichome birefringence-like 36 which may be involved in the synthesis and deposition of cellulose in SCWs; presumably by affecting the esterification state of pectic polymers (Bischoff et al., 2010). Importantly, a group of up-regulated genes encoding NAC010 domain proteins, involved in the regulation of SCW thickening, were found in both roots and stems. An ABA-responsive *PtrMYB167* transcription factor, involved in the regulation of SCW biogenesis, was also found to be expressed in both roots and stems (Chai et al., 2014).

### Immunolocalization of Cell Wall Components

**Figure 5:**
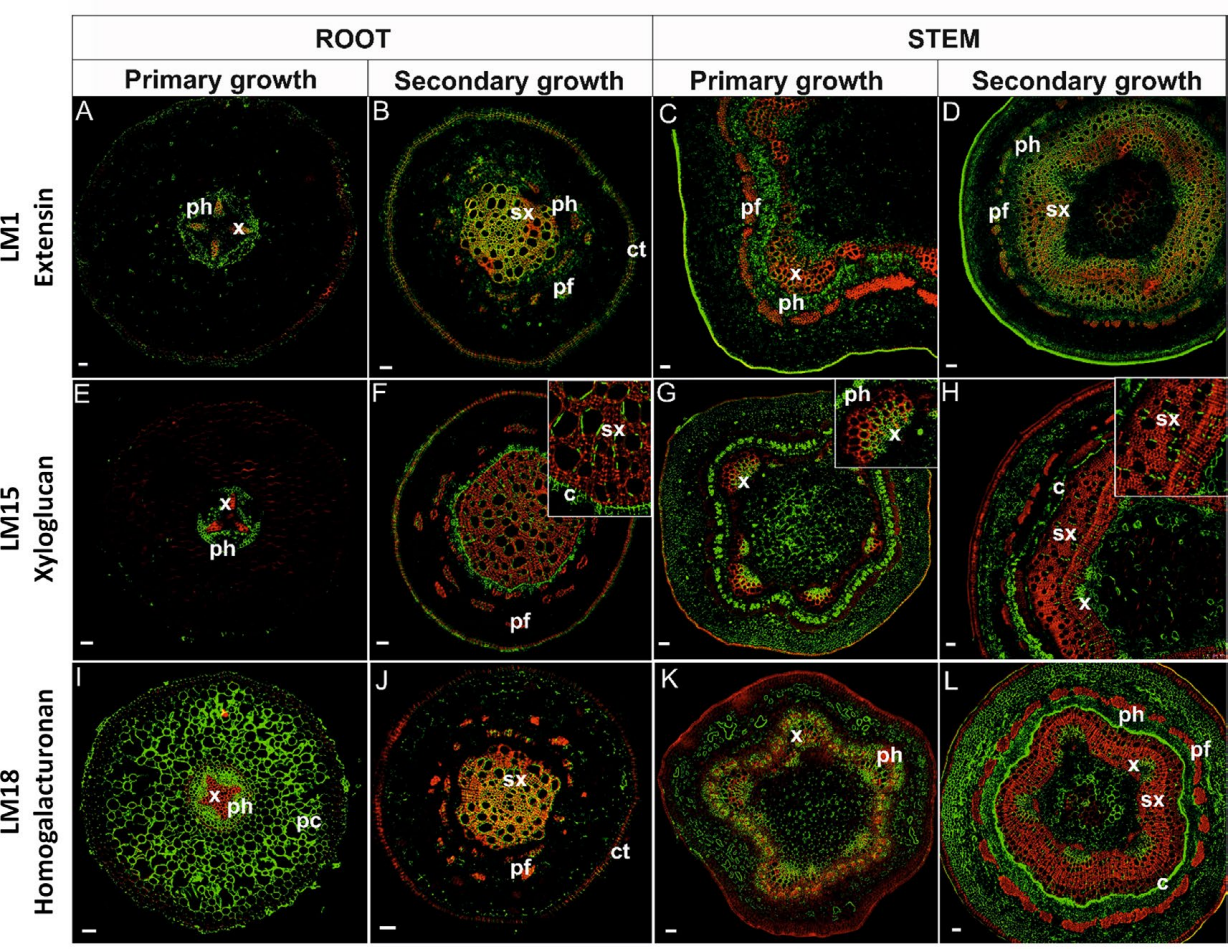
Immunolocalization of extensins **(A**–**D)**, xyloglucan **(E**–**H)**, homogalacturonan **(I**–**L)**, and their co-localization with lignins in stems and pioneer roots during different developmental stages. [x, primary xylem; ph, phloem; sx, secondary xylem; pf, phloem fibers; c, cambium; ct, cork tissue (phellem); pc, parenchyma cortex]. Bars 50 µm.

**Figure 6:**
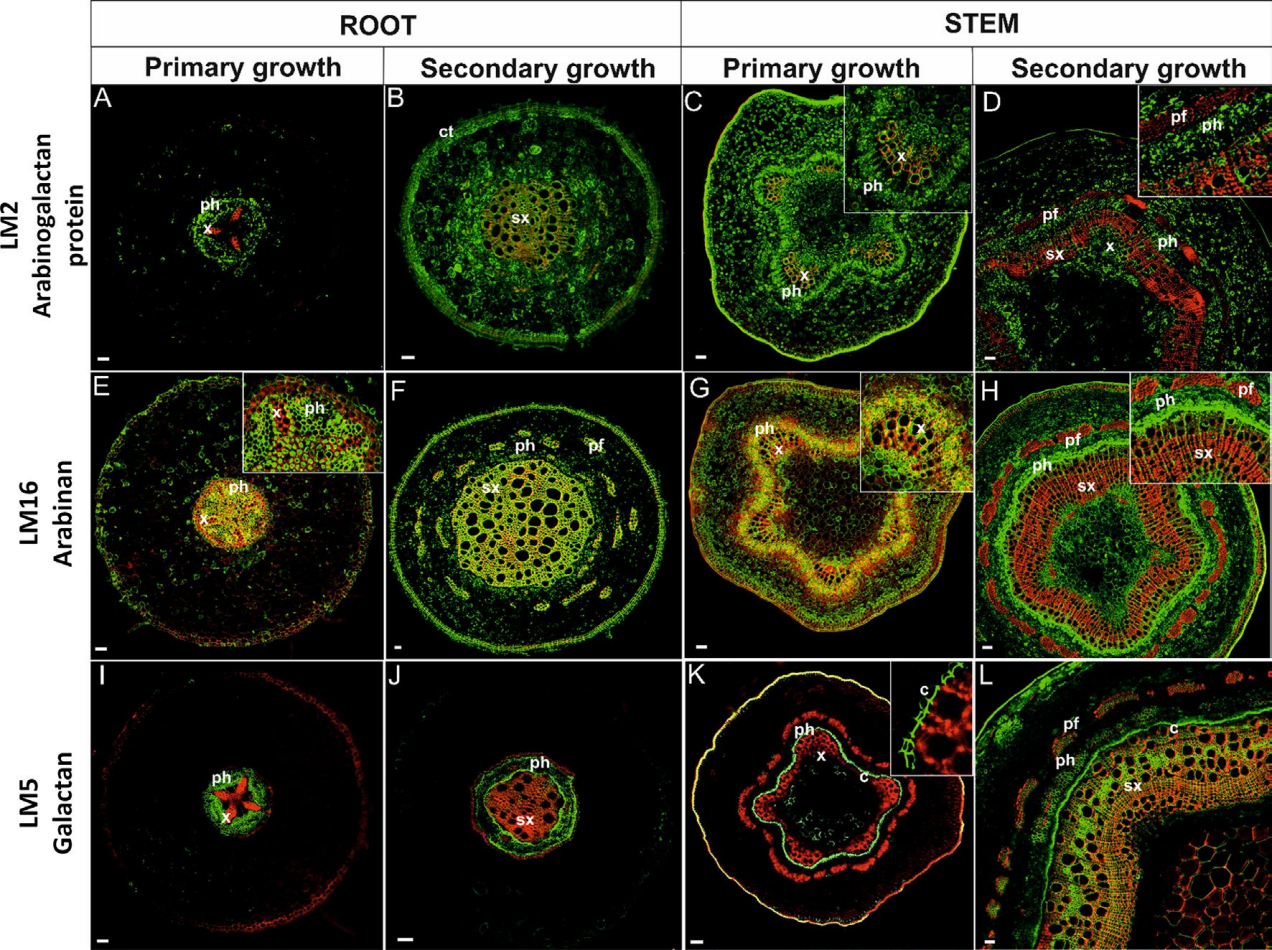
Immunolocalization of arabinogalactan proteins **(A**–**D)**, arabinan **(E**–**H)**, galactan **(I**–**L)**and their co-localization with ligninsin in pioneer roots and stem during different developmental stages. [x, primary xylem; ph, phloem; sx, secondary xylem; pf, phloem fibers; c, cambium; ct, cork tissue (phellem)]. Bars 50 µm.

Immunolocalization studies were conducted to determine the impact of differential gene expression on the localization of various cell wall components. Selective antibodies against cell wall components were used for determining the localization and visualization of cell wall polysaccharides and select proteins during root and stem development. The localization of lignin was also determined by its autofluorescence. Lignins, which are an important component of the cell wall and convey rigidity, were located mainly in xylem vessels, xylem fibers, and also phloem fibers (**Figures 5**, **6** and **Table 1**).

**Table 1:** Stele tissue specific distribution of cell wall epitopes in *Populus trichocarpa* pioneer roots and stems during their primary and secondary growth.

		Root				Stem				
		Primary tissues		Secondary tissues		Primary tissues		Secondary tissues		
		*Phloem*	*Xylem*	*Phloem*	*Xylem*	*Phloem*	*Xylem*	*Cambium*	*Phloem*	*Xylem*
LM1	Anti-extensin	+	++	+*	+++	+	+		*	++
LM2	Anti-arabinogalactan-protein	++		+*	++	+	+	±	+	+
LM5	Anti-(1-4)-β-D-galactan	++		+	±	±	±	+++	+*	++
LM15	Anti-xyloglucan			*	±	+	+	+	+	±
LM16	Anti-processed arabinan/RG-I	++		*	++	++	++	++	++	±
LM18	Anti-homogalacturonan	+		*^(±)^	++	+	++	+++	++	

(++/+++), strong binding; (+), binding; (±), weak binding; (−), lack of binding; *, binding in phloem fibers.

Extensins are HRGPs that are secreted as rod-like monomers with limited flexibility. An anti-extensin antibody, which recognizes an epitope that is carried by a range of HRGPs of the extensin class (Smallwood et al., 1995), was used in the present localization study. The LM1 antibody exhibited different labelling patterns across the different tissues examined. In primary growth of *roots* (**Figure 5A**), HRGPs were localized mainly in xylem TEs and phloem cells. In cross-sectioned roots with secondary growth (**Figure 5B**), the antigen was localized also in all lignified and secondary tissues, both secondary xylem and phloem (including phloem fibers); as well as in phellem. Moreover, the signal was greater as lignification in the tissues increased (**Table 1**). In *stems*, the LM1 antigen was localized in primary xylem and phloem cells (**Figure 5C**) and strongly in lignified SCWs (**Figure 5D**, **Table 1**). A positive reaction was also observed in phellem tissues.

Xyloglucan was localized using an LM15 antibody that recognizes the XXXG motif of xyloglucan and it is also capable of cross-binding to a single galactosyl residue (Marcus et al., 2008). Xyloglucan in *roots* in primary growth was not observed in the TEs of xylem, but rather only in procambial tissue below the phloem and in the cells close to the xylem pole (**Figure 5E**). In roots with secondary growth, however, xyloglucan labeling was present in the phellem tissue, cambial cells, and to a lesser extent in TEs; especially those with SCW thickening (**Figure 5F**). A weak signal was present in phloem fibers. In *stems* in primary growth, xyloglucan was present in the xylem of vascular bundles; mostly in the protoxylem, developing phloem, and the hypodermis (**Figure 5G**). In stems with secondary growth, a strong xyloglucan signal was detected in the primary xylem, cambial cells, and phloem (except phloem fibers). A positive signal was present in some walls of TEs with SCW thickenings (**Figure 5H**).

Among the three known classes of pectin polysaccharides in plant cell walls, the localization of HGs was examined. An LM18 antibody, which has a preference for partially methyl-esterified HG epitopes but can also bind to un-esterified HG (Verhertbruggen et al., 2009), was used in the localization studies. The use of the LM18 antibody enabled the localization of the deposition of HGs, as well as partially methyl-esterified pectin epitopes in cell walls. HGs were abundantly observed in the cell walls of cortical parenchyma cells of *roots* in primary growth and the cell walls of all un-lignified tissues (**Figure 5I**). Remarkably, there was a lack of signal in the cells of the hypodermis. In roots with secondary growth, HGs were observed mainly in the cell walls of secondary xylem cells (**Figure 5J**). The anti-HGs antibody failed to indicate any differential labelling in *stems*, however, labeling was observed in the cell walls of all primary tissues: primary phloem, primary xylem, and parenchyma tissue (**Figure 5K**). In stems with secondary growth, HGs were again evident in PCWs, cells in the cambial zone, phloem, and within primary xylem (**Figure 5L**). Phloem fibers and secondary xylem cells did not exhibit HGs (**Figure 5L**). Importantly, HGs did not co-localize in lignified cell walls (**Figure 5L**).

An LM2 antibody that recognizes a carbohydrate epitope containing β-linked glucuronic acid, allowing the antibody to recognize AGPs (Smallwood et al., 1996; Yates et al., 1996), was used in the current study. AGPs in *roots* in primary growth were localized to cells in the stele but were absent in the cortex (**Figure 6A**). The antibody was localized to the PCW of procambial and phloem tissues but not to xylem. In roots with secondary growth (**Figure 6B**), AGPs labeling was observed in almost the whole cross-section of the root, including both primary and secondary lignified cell walls. In *stems* in primary (**Figure 6C**) and secondary growth (**Figure 6D**), arabinogalactan epitopes were observed in all tissues; including lignified TEs of xylem. Only mature phloem fibers lacked arabinogalactans.

**Figure 7:**
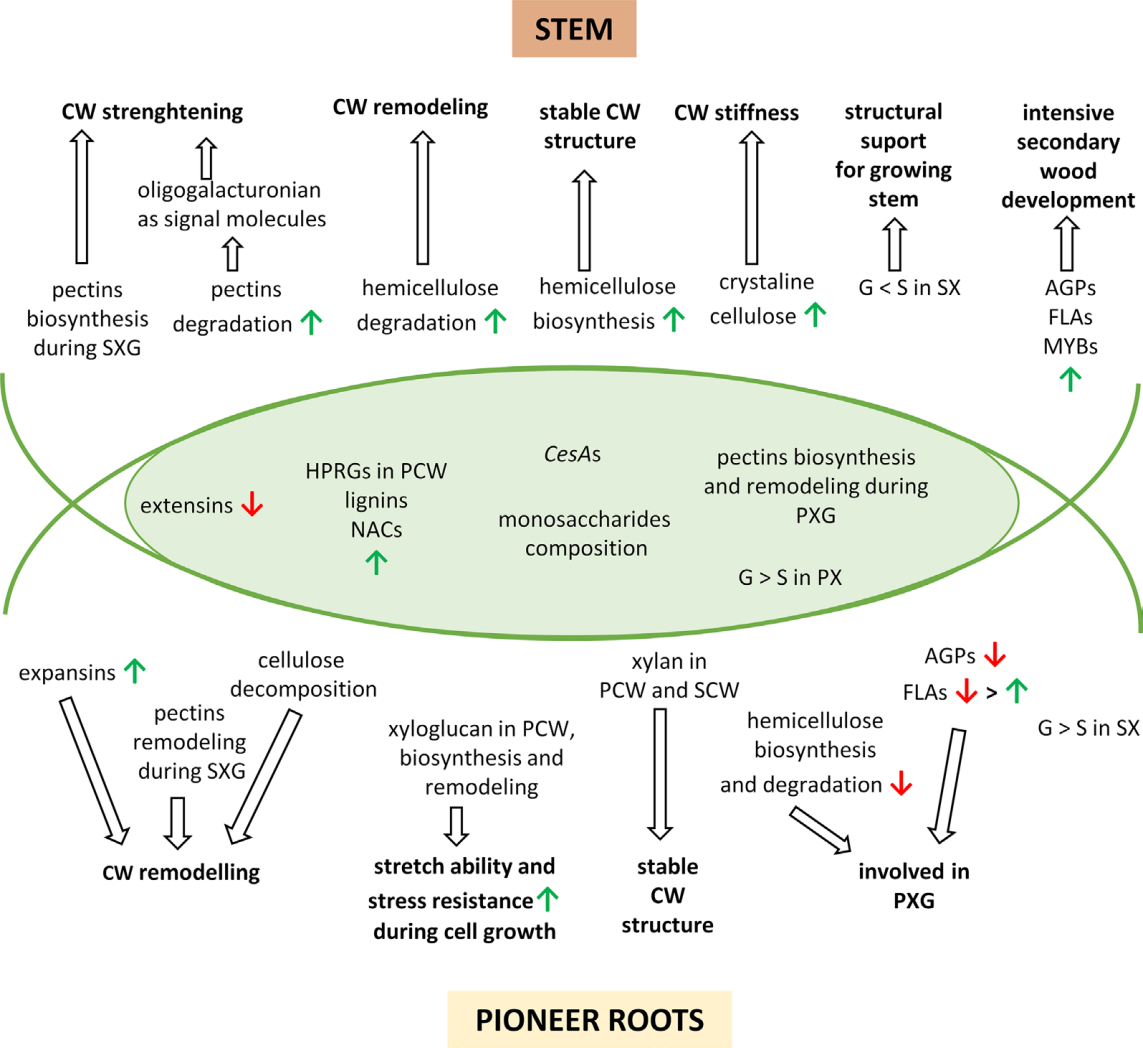
A simplified model to describe the major changes in developing pioneer roots and stems of *Populus trichocarpa*. The overlapping region corresponds to common processes in pioneer roots and stems. The upward green arrows show up-regulated features and the downward red arrows show down-regulated features. CW, cell wall; PCW, primary cell wall; SCW, secondary cell wall; PX, primary xylem; PXG, primary xylem formation; SX, secondary xylem; SXG, secondary xylem formation; G, guiacyl units in lignins; S, syringyl units in lignins; AGPs, arabinogalactan-proteins; FLAs, fasciclin-like arabinogalactan-proteins.

The complexity of arabinan polysaccharides deposition was analyzed using an LM16 antibody that recognizes a processed arabinan/RG-I epitope associated with arabinans. The enzymatic activity of arabinofuranosidase can generate arabinans; resulting in the loss of arabinosyl residues (Verhertbruggen et al., 2009). Arabinan in *roots* in primary growth was present in all unlignified cell walls of the stele, but not in the cell walls of primary xylem cells (**Figure 6E**). Arabinan localization in roots with secondary growth was observed in the phellem, secondary xylem, and secondary phloem fibers but absent in other tissues (**Figure 6F**). In *stems* in primary growth, arabinan was present in parenchyma cells, phloem, and to some extent also in xylem tissue (**Figure 6G**). In stems with secondary growth, however, a strong amount of labeling was observed in cambial cells and their derivative phloem initials. Arabinan was also present in primary xylem and a weak signal was observed in secondary xylem (**Figure 6H**).

Distinctive labeling patterns were observed for galactan. The LM5 antibody, which recognizes a linear tetrasaccharide in (1-4)-β-D-galactans (Jones et al., 1997), was used in the localization studies. Galactans in *roots* in primary growth were localized in the phloem tissues (**Figure 6I**). No galactan labeling was observed in the cortex and xylem. In roots exhibiting the onset of secondary development, galactan was observed in the phloem and lightly in secondary xylem tissue (**Figure 6J**). No other tissue exhibited galactan labeling. In *stems* in primary growth (**Figure 6K**), galactan exhibited a far greater localization in cambial tissue and the first phloem derivatives. Only a slight amount of immunostaining was observed in the primary xylem. Labeling was also observed in the hypodermis. There was a significant change in galactan labeling in stems with secondary growth (**Figure 6L**). A strong degree of galactan labeling was observed in the secondary xylem, cambium, and phloem; including phloem fibers.

### Quantification of Cell Wall Components

#### Cell Wall Monosaccharide Composition

Many similarities were observed in the monosaccharide composition of pioneer roots and stems (**Figure 3C**). A very low ratio of rhamnose and fucose was detected in both organs, and the level of arabinose and glucose decreased with xylem development. In contrast, xylose increased in the cell walls of both organs and was the predominant cell wall monosaccharide in cell walls throughout the entire process of xylogenesis. A higher amount of galactose was detected, however, in pioneer roots than in stems.

#### Crystaline Cellulose Content

Slight growth of cellulose content was observed in the secondary growth segment of pioneer roots (19%) comparing to the apical meristem (18%) and primary roots (18%) (**Figure 3D**). While in the stem, cellulose content increased along with the progress of xylem development (**Figure 3D**). The greatest increase was observed in isolated mature xylem (29%) comparing to stem in primary (15%) and secondary growth (19%). Moreover, the lowest cellulose content was measured in stem in primary growth, while in the secondary structure of roots and stems, cellulose amount was on a very similar level.

#### Lignin Composition

A significant difference was observed between lignin composition in pioneer roots and stems, however, a very low level of H fraction was detected in both organs (**Figure 4E**). No changes in the ratio of the monolignols were observed in roots. G was the dominant form (60–67%) and S was the second dominant form (32–38%) in all stages of root development. In contrast, dynamic changes were observed in stems at different stages of xylem development. The highest content of the G form (53%) was observed in stems at the initial stage (PS1) of xylogenesis. The level of the G form, however, decreased (41%) during the second stage (PS2) of xylogenesis and remained at a similar level (39%) in mature xylem PS3. In contrast, the level of the S form increased during xylogenesis from 45% in PS1 to 57% and 61% in PS2 and PS3, respectively; thus becoming the predominant form in the cell walls of mature xylem.

## Discussion

Xylogenesis is one of the most characteristic genetically programmed processes of cell differentiation in higher plants. Moreover, it is a typical example of developmentally regulated PCD. To date, no large *in vivo* analyses of xylogenesis have been conducted. Considering the number of different stages of xylogenesis, special attention should be paid to the formation of the lignified SCW and the characteristic wall thickenings of TEs. Lignification is an irreversible process which is usually preceded by the deposition of cellulose and non-cellulosic matrix components, such as hemicelluloses, pectins, and cell wall proteins (Fukuda, 1996; Turner et al., 2007; Barros et al., 2015). Thus, one aspect of the analysis should consider the changes in cell wall composition that occur during the course of xylogenesis (PCWs of developing xylem TEs *vs.* the SCWs of developing vessels and fibers) and determine if composition differs between roots and the aerial parts (stems) of a tree. In the current study, a general overview of changes occurring in cell walls during xylogenesis in pioneer roots and stems of *P. trichocarpa* was presented. Xylogenesis requires the coordinated expression of many genes and our study demonstrates that different regulatory mechanisms often underlie primary and SCW development in studied organs.

HRGPs are the major structural proteins in cell walls and extensins are well-characterized proteins belonging to this group (Lamport et al., 2011). Results of the current study indicate that they are mainly associated with PCW development in both pioneer roots and stems, although to a lesser extent in stems. Even though the expression of genes encoding extensins is down-regulated during plant development, they remain a significant component of secondary tissues. Extensins were localized to both primary and secondary xylem cells in pioneer roots and stems, moreover, the signal was stronger as the level of lignification increased. Another group of cell wall structural proteins that play an important role in plant cell growth and developmental processes are expansins (Cosgrove, 1998), which, may be involved in cell wall modifications during xylogenesis in roots of *P. trichocarpa* but not in stems.

AGPs are involved in many processes in plants including plant growth and development, root growth and development, xylem development, and PCD (Motose et al., 2004). The current study indicates that AGPs are mainly involved in the early stages of xylem development in roots and in the later stages of cell wall development in stems. Interestingly, AGPs in roots in primary growth were not localized in xylem cells; however, in roots in secondary growth, they were found in both primary and secondary lignified cell walls. In contrast, AGPs were observed in all tissues of stems in primary and secondary growth, including lignified TEs. Although the exact role of FLAs has not been fully defined, some have been linked to SCW development (MacMillan et al., 2010; Jun and Xiaoming 2012; Zang et al., 2015). The current analysis confirmed that FLAs are associated with SCW development during both the primary and secondary xylem formation in pioneer roots, as well as in secondary xylem development in stems.

Carbohydrates are the main components of the PCW, and among them, pectins are the most abundant. In addition to pectins, there is also a high content of cellulose and hemicelluloses that are present in PCWs (Plomion et al., 2001; Caffall and Mohnen, 2009). In contrast, cellulose, with significantly fewer hemicelluloses and pectins, is the main component of SCWs (Weng et al., 2008; Fincher, 2009). In the current study, a coordinated expression of genes encoding cell wall components was observed; indicating that temporal changes in gene expression regulate primary and SCW formation in response to specific genetically defined programs of cell development.

Mellerowicz and Gorshkova studies (2012) reported that the primary and secondary walls of *Populus* xylem differ in their composition of carbohydrates. The PCWs predominantly contain pectins (HG and RG-I) and a high content of hemicelluloses (xyloglucan, arabinoxyloglucuronoxylan, mannan) and cellulose (Plomion et al., 2001; Caffall and Mohnen, 2009; Mellerowicz and Gorshkova, 2012). On the other hand, the layers of SCWs are rich in cellulose with significantly lower levels of hemicelluloses (glucuronoxylan) and pectins (Weng et al., 2008; Fincher, 2009; Mellerowicz and Gorshkova, 2012). The rearrangements that occur in the cell wall during the transition from primary to secondary growth indicate that the process is highly regulated by molecular mechanisms. In our study, numerous genes encoding CesA were identified that are orthologues of *A. thaliana CesA*s that have been reported to be significantly up-regulated in developing xylem (Aspeborg et al., 2005; Suzuki et al., 2006). Our data indicate a variable expression pattern for different *CesA* genes, suggesting that they are involved in primary and/or secondary xylem formation. However, the *Ces7A-like* gene, which is typically associated with SCW development, was down-regulated during secondary xylem development. On the other hand, the expression of *PtiCesA3-1*, which is associated with PCW development, was up-regulated during secondary growth of roots. These results suggest that some *CesA* genes that have been previously considered to be specific to either primary or SCW development may play a more general role in cell wall development. Similar observations were reported by Sundell et al. (2017) in a study of wood formation in stems of *Populus tremula.* In addition to high levels of cellulose biosynthesis occurring during primary and SCW development, cellulose decomposition also occurs during the remodelling of the cell wall during the course of xylem maturation and lignification in roots. Interestingly, more genes related to cellulose biosynthesis were found to be up-regulated in stems than in roots. These data suggest that a greater accumulation of cellulose occurs in the xylem cell wall of stems, which was also confirmed when cellulose content in cell walls was measured.

Hemicellulose synthesis-related genes were identified in both roots (xylan and xyloglucan) and stems (xylan). Their expression pattern suggests that hemicelluloses are synthesized during both primary and SCW formation. *IRX9* and *IRX14* genes with different patterns of expression were identified in pioneer roots. *IRX9* was up-regulated during both the primary and secondary xylem formation, while *IRX14* was down-regulated in roots during secondary growth. Ratke et al. (2015) reported that IRX9 and IRX14 are both parts of a xylan synthase complex during secondary growth in hybrid aspen. Our results suggest, however, that IRX14 is only involved in xylan biosynthesis during primary growth. Xyloglucan was not observed in xylem TEs of roots with primary growth and was present to a lesser extent in roots with secondary growth, especially within TEs containing SCW thickenings. In contrast, xyloglucan was localized to primary xylem in stems exhibiting both primary and secondary growth. Compared to roots, a positive signal appeared in some walls of TEs that had thickened during differentiation. Similar to a previous observation by Bourquin et al. (2002), it is possible, however, that the signal is derived from xyloglucan located in the PCW. Hemicellulose degradation is also an integral part of cell wall remodelling (Mellerowicz and Sundberg, 2008; Minic, 2008). In roots, our study indicates that hemicelluloses are broken down only during the primary xylem differentiation; whereas in stems, hemicelluloses are degraded during both primary and secondary development. These observations suggest that a continuous metabolism of hemicelluloses occurs, which may be due to more extensive modification of cell wall carbohydrates resulting from the greater level of lignification that occurs in stems relative to roots.

Data from the current study suggest that the early stage of pectin biosynthesis in roots is completed prior to secondary growth begins. In stem tissues, however, pectin biosynthesis continues even after the second stage of xylogenesis is initiated. HG and RG-I (galactan and arabinan) are pectins that occur in PCW layers in *Populus* xylem (Mellerowicz and Gorshkova, 2012) and HG is the major cell wall pectin (O’Neill et al., 1990; Mohnen et al., 2008). HGs were observed in the cell walls within cortical parenchyma cells and all un-lignified tissues of roots in primary growth. In roots in secondary growth, however, HGs were mainly present in secondary xylem cells; possibly in the PCW layers of these cells. In stems in primary growth, HGs were localized in the cell walls of all tissues, whereas in stems with secondary growth, HGs were only located in the PCWs of cambial zone cells, phloem, and primary xylem. Galactan was localized in the cell walls of phloem cells in roots in primary and secondary growth, and slightly in the cell walls of secondary xylem tissue. In stems with primary growth, an accumulation of galactan was observed in cambial cells and a slight amount in primary xylem cells. In stems with secondary growth, however, a strong galactan signal was observed in the secondary xylem, cambium, and phloem. Similar to galactan, arabinan was observed in all un-lignified cell walls in roots with primary growth, but not in already lignified primary xylem. In roots in secondary growth, arabinan was localized to phellem cells, secondary phloem fibers, and secondary xylem; most likely in the PCW layers. In stems in primary growth, arabinan occurred mainly in phloem and to some extent xylem tissue. In stems with secondary growth, however, a stronger signal was observed in cambial cells and phloem initials; as well as in primary xylem and a little in secondary xylem. Remodeling of cell walls in pioneer roots is associated with pectin degradation, pectin de-esterification, acetylation, and de-methylation; which occurs during both the primary and secondary xylem formation. In contrast, pectins in stems are modified through de-methylation and degradation by pectinase.

The level of glucose and arabinose decreased in roots and stems with secondary growth, which may be explained by the fact that they construct hemicelluloses and pectin RG-I (arabinose) that are typically found in PCWs (Hoch, 2007; Mellerowicz and Gorshkova 2012). In contrast, the level of xylose increases; which is typical for the hemicelluloses (xylan) and pectins (glucuronoxylan) in SCWs (Mellerowicz and Gorshkova, 2012).

The majority of genes related to lignin biosynthesis were up-regulated during both the primary and secondary xylem formation, however, a greater level of up-regulation was observed in their expression during secondary growth in both roots and stems. It is possible that enzymes associated with the early steps of the monolignol pathway are also involved in the biosynthesis of other phenylpropanoids (Koutaniemi, 2007; Sundell, 2017). Moreover, the level of lignin increased with the development of secondary growth in pioneer roots and stems. Lignins were located mainly in the cell walls of xylem vessels, xylem fibers, and phloem fibers. SCWs of TEs in angiosperm wood contains mainly G-units, while sclerenchyma fibers contain both G- and S-units. Similar to what was observed in our study, the general proportions of H/G/S lignins in angiosperms in TE-rich tissues are 0-8/25-50/46-75 (Ek et al., 2009). Reduced expression of *COMT* genes causes decreased formation of S-units in most cases (Christensen and Rasmussen 2019). Therefore, the constant level of S-units in pioneer roots may be explained by rather stable over-all expression of genes encoding COMT. However, G was the dominant form in roots, where the amount was as high as 60–67% and did not significantly change during the course of xylogenesis. Since there is no need for additional support in the roots, there is no significant increase of sclerenchyma fibers containing G- and S-units. In contrast, the S/G ratio increased in stems during secondary growth (PS2). Increasing expression of genes encoding COMT in PS2 and PS3 is accompanied by increased level of S-units in stems. Moreover, it is possible that this is due to the down-regulation in *PAL* expression in PS2 (Wang et al., 2014) and results in the increasing amount of sclerenchyma fibers produced in stem tissues to provide structural support of the growing stem.

NAC domain and MYB transcription factors act as master switches regulating gene expression during secondary wall biosynthesis (Zhong and Ye, 2014). In our study, genes encoding NAC domain proteins were mostly up-regulated in roots and stems during xylogenesis; suggesting that they may be involved in the regulation of genes involved in cell wall biosynthesis and cell wall modifications. While *MYB* TFs in pioneer roots were both up- and down-regulated, they were mostly up-regulated in stems. Consequently, it is plausible that this up-regulation increases the expression of genes in pathways involved in SCW formation which in turn may be responsible for the greater development of secondary xylem in stems relative to roots.

Although many studies of xylogenesis have been conducted in stems, much less is known about xylem formation in roots. Our present study provides a detailed, comprehensive description of the expression of genes during cell wall developments and cell wall modifications occurring during xylogenesis of pioneer roots and stems in *P. trichocarpa.* Interestingly, the majority of DEGs in pioneer roots *vs.* stems during xylogenesis was very different, with only approximately 10% of the DEGs showing commonality to both organs. Despite this major difference, however, many characteristics of xylogenesis are similar; such as increasing expression for HRGPs in primary call wall, decreasing expression for extesins, differentiated expression of genes encoding CesAs and increasing lignins synthesis with G-units being dominant over S-units in primary xylem. Also similar pattern of pectins biosynthesis and remodeling during primary xylogenesis was observed in both roots and stems. Moreover, the composition of monosaccharides in both organs is also very similar. For other components, however, the timing of the up- or down-regulation is different due to diverse role of both organs and differences in environment under- and aboveground. For example, AGPs and most FLAs are only involved in primary xylogenesis in roots, hemicelluloses are only degraded in the PCW of roots; whereas, these features are expressed continuously throughout all stages of xylogenesis in stems due to intensive cell wall remodeling and secondary wood development. Some processes appear to be unique to one organ, e.g. the synthesis of expansins and cellulose degradation in roots leading to cell wall remodeling during secondary xylogenesis. Others are more intensive in one organ, such as the level of pectin remodeling that occurs in roots. In roots, xylan helps to stabilized the structure of cell walls, and biosynthesis and remodeling of xyloglucan ensure stretch ability and stress resistance during cell growth. While in stems, pectins biosynthesis and signaling molecules arising during pectins degradation lead to cell wall strengthening. Increasing biosynthesis of hemicellulose provides stable cell wall structure, while expanded level of crystalline cellulose ensures cell wall stiffness. Moreover, predominance of G-units over S-units in lignins in secondary xylem provides structural support for the growing stem (**Figure 7**).

The present study provides the first comprehensive structural and molecular analysis, including an analysis of gene expression, of the differentiation of TEs (vessels) and supporting elements (fibers) within xylem in pioneer roots in comparison with stems of *P. trichocarpa*. The current and previously reported information clearly reveals the great complexity of molecular mechanisms underlying the cell wall formation and modifications that occur during xylogenesis. Our research increases the knowledge and improves understanding of the cell wall development in under- and aboveground tree organs. Efforts to breed new tree varieties with higher yield and better wood quality will not be successful without recognizing and understanding the complicated transcriptional network underlying wood development.

## Material and Methods

### Plant Material and Experimental Design

All experiments were performed on seed-grown *P. trichocarpa* (Torr. & A. Gray ex Hook.) growing in rhizotrons at the Institute of Dendrology, Polish Academy of Sciences in Kórnik, Poland (52°14’40” N 17°06’27” E). Seeds were obtained from the FLORPAK Młynki Seeds Store, Poland. Seedlings were initially grown in a plant growth chamber (Conviron GR96) at 18°C day/14°C night and a 16 h day/8 h night photoperiod. After 3 months (in April), plants were transferred into rhizotrons. Roots were grown in transparent-walled chambers filled with natural soil with shoots extending from the top into the air. The rhizotrons (50x30 cm) were constructed of two transparent polycarbonate plates held 3 cm apart by thick-walled plastic tubing to provide adequate growing space. Waterlogging was avoided by installing a drainage hole in the bottom of each rhizotron that permitted soil aeration and drainage of excess water.

Material was collected in July, in the middle of vegetative season. Pioneer roots in all of the experiments were divided into the following segments corresponding to their developmental stage: 0-2 cm—root tips with apical meristem (PR1); 4–6 cm—primary growth (PR2); and 13–16 cm—secondary growth (PR3). Similarly, stems were also sampled based on developmental stages: 0–2 cm—apical meristem with primary growth (PS1); 20–25 cm—secondary growth (PS2), and 40–45 cm—isolated secondary xylem (PS3) (**Table 2**). Root tips were treated as a negative control for the process of xylogenesis, since the process of xylogenesis is undetectable in root tips, while isolated secondary xylem served as a positive control for the xylogenesis process.

**Table 2:** Experimental design describing sampling of *Populus trichocarpa* pioneer roots and stems.

**Root**	PR1	Root tips with apical meristem
	PR2	Primary growth
	PR3	Secondary growth
**Stem**	PS1	Apical meristem with primary growth
	PS2	Secondary growth
	PS3	Isolated secondary xylem

### Microarray Analysis

Total RNA was extracted from three biological replicates of each developmental stage of roots and stems as described above (see section *Plant Material and Experimental Design*) using an RNeasy Plant Mini kit (Qiagen, USA). RNA quantity and quality were assessed using a NanoDrop1000 (Thermo Fisher Scientific Inc., USA). cRNA synthesis and microarray hybridization to an Affymetrix GeneChip Poplar Genome Array (A-AFFY-131) were performed according to the provided Affymetrix protocol. The microarray experiments were performed at the Laboratory of Microarray Analysis (Institute of Biochemistry and Biophysics, Polish Academy of Sciences, Warsaw, Poland). A complete microarray dataset was submitted to the Gene Expression Omnibus database (accession number GSE126842). The raw image data from a total of three A-AFFY-131 arrays were normalized with Robust Multi-Array Average (RMA). The normalized data were statistically analyzed using GeneSpringGX7 13.1 (Agilent Technologies Inc., USA) software. Data were subjected to a one-way ANOVA with a Benjamini-Hochberg corrected p-value cut-off = 0.05. The analysis was limited to genes with statistically significant differences in expression level and a fold difference equal to or larger than 2 between PR1 and PR2/PR3 in the pioneer root samples and between PS1 and PS2/PS3 in stem samples.

### Immunolocalization of Cell Wall Components

Root and stem segments were sectioned (30 µm) using a vibratome (Leica VT 1200S, Leica Biosystems, Germany) and immediately fixed for 1 h in 0.5% glutaraldehyde with 4% formaldehyde in phosphate buffered saline (PBS) buffer (0.1 M, Sigma-Aldrich, USA). The material was rinsed with PBS and then sections were blocked with 2% bovine serum albumin (BSA, Sigma-Aldrich, USA) in PBS for 20 min. The sectioned samples were subsequently treated for 2.5 h at room temperature (RT) with different IgG Rat Primary Antibodies (PlantProbes; LM1—for extensins, LM2—for AGPs, LM5—for galactan, LM15—for xyloglucan, LM16—for arabinan, or LM18—for HG) that were diluted 1:10 with 0.2% BSA/PBS. The samples were rinsed five times in PBS, and then incubated at RT for 1.5 h with Goat Anti-Rat IgG (H+L) Secondary Antibody, Alexa Fluor^®^ 488 Conjugate (Thermo Fisher Scientific Inc., USA), diluted 1:100 with BSA/PBS. After rinsing in PBS, the material was mounted in Prolong Gold (Life Technologies, Thermo Fisher Scientific Inc., USA). Results of the immunolocalization assay were recorded with a Leica TCS SP5 II confocal microscope (Leica Biosystems, Germany) using lasers: 405 diode emitting light at wavelengths of 405 to observe autofluorescence of lignins and argon laser emitting light at wavelengths 488 to observe fluorescence fluorochrome Alexa 488 (secondary antibodies using in IC reactions). Lignin autofluorescence was also characterized at the same time the immunolocalization studies were conducted. At least five root and stem segments were harvested from each developmental category for the analysis of each tested antibody. Incubations without primary antibodies were used as a negative control. No detectable results were obtained with the negative controls.

### Quantification of Cell Wall Components

#### Cell Wall Alcohol Insoluble Residue (AIR) Isolation

Four independent samples of cell walls were extracted from each studied developmental stage of roots and stems sampled as described above (see section *Plant Material and Experimental Design*). Plant tissue was frozen in liquid nitrogen and ground in a ball mill (Retsch, Germany) to a fine powder. A total of 60 mg of tissue was extracted with 70% aqueous ethanol and chloroform/methanol (1:1 v/v) solution resulting in an AIR fraction. Starch was removed by enzymatic digestion with α-amylase and pullulanase. The material was air-dried and stored until further processing (Foster et al., 2010).

#### Cell Wall Monosaccharide Composition

Two mg of AIR were hydrolyzed with 2 M trifluoroacetic acid for 90 min at 121°C and the resulting monosaccharides were reduced to their corresponding alditols with sodium borohydride in 1 M ammonium hydroxide. The acetylation of the alditols to alditol acetates was performed using acetic anhydride and pyridine. Alditol acetates were extracted with ethyl acetate/water (Foster et al., 2010). Samples were diluted with acetone and analyzed using GC/MS. Myo-inositol was used as an internal standard. Cell wall monosaccharide composition was measured for four replicates per each studied object.

#### Crystaline Cellulose Content

Two mg of AIR were hydrolyzed with 2 M trifluoroacetic acid for 90 min at 121°C. The resulting pellet containing crystalline cellulose was treated with Updegraff reagent (acetic acid:nitric acid:water, 8:1:2 v/v) and heat at 90°C for 30 min. Crystalline cellulose was then completely hydrolyzed into glucose with 72% sulfuric acid (Foster et al., 2010). Samples were diluted with water and glucose content of the supernatant was assayed using the colorimetric anthrone assay. Glucose (and hence crystalline cellulose content) was calculated based on the absorbance at 625 mm compared to the glucose standard curve established on the same plate (Foster et al., 2010). Crystaline cellulose content was measured for four replicates per each studied object. 

Lignin Composition

Two mg of AIR were subjected to thioacidolysis for 4 h at 100°C in a nitrogen atmosphere with 2.5% boron trifluoride diethyl etherate (BF^3^), and 10% ethanethiol (EtSH) solution in dioxane. Samples were cleaned-up with ethyl acetate/water and the ethyl acetate layer was subsequently collected and air-dried. For the TMS derivatization, ethyl acetate, pyridine, and N,O-bis(trimethylsilyl) acetamide were added to each tube and incubated for 2 h at 25°C (Foster et al., 2010). Samples were diluted with acetone and analyzed with GC/MS. Lignin composition was measured for four replicates per each studied object.

#### Gas Chromatography/Mass Spectrometry Analysis

The GC/MS analysis was performed using an Agilent 7890 A gas chromatograph (Agilent Technologies, USA) connected to a Pegasus 4D GCxGC-TOFMS Mass Spectrometer (Leco, USA). A DB-5 bonded-phase fused-silica capillary column (30 m length, 0.25 mm inner diameter, 0.25 µm film thickness) (J&W Scientific Co., USA) was used for separation. The GC oven temperature program was as follows: 2 min at 70°C, raised by 10°C/min to 300°C and held for 10 min at 300°C. The total time of GC analysis was 36 min. Helium was used as the carrier gas at a flow rate of 1 ml/min. One microliter of each sample was injected in splitless mode. The initial injector temperature was set at 40°C for 0.1 min and was subsequently raised by 60°C/min to 350°C. The septum purge flow rate was 3 ml/min, which was turned on after 60 s. The transfer line and ion source temperatures were set to 250°C. In-source fragmentation was performed with a 70 eV energy. Mass spectra were recorded in the mass range of 35–650 m/z.

Data acquisition, automatic peak detection, mass spectrum deconvolution, retention index calculation, and library searches were performed using LECO ChromaTOF Software (Leco, USA). To eliminate retention time (Rt) shift and to determine the retention indexes (RI) for each compound, an alkane series mixture (C-10 to C-36) was injected into the GC/MS system. The metabolites were automatically identified using a library search (NIST library). The analyte was considered as identified when it passed a quality threshold of a similarity index (SI) above 700 and a matching RI ± 10. Artefacts [alkanes, column bleed, plasticizers, N-methyl-N-(trimethylsilyl)trifluoroacetamide, and reagents] were identified analogously and excluded from further analyses. Unique quantification masses for each component were specified and the samples were reprocessed to obtain accurate peak areas for the deconvoluted components. The obtained profiles were normalized against the sum of the chromatographic peak area (using the total ion chromatogram (TIC) approach).

### Statistical Analysis

Statistical analyses of the differences in crystalline cellulose content between the studied fragments of pioneer roots and stems (ANOVA and Tukey’s HSD test, p < 0.05) were performed using Statistica 12.0 Software (StatSoft Poland Inc., USA).

## Data Availability Statement

The datasets generated for this study can be found in the Gene Expression Omnibus database: accession number GSE126842.

## Author Contributions

KM-S and NW collected material and performed the analyses with contributions from AL, AK-M, and JM. AB-Z conceived the original concept and research plan, supervised the experiments, and provided funding. KM-S and AB-Z analyzed the data. KM-S wrote the article with critical comments provided by AL and AB-Z. All authors discussed the results, read, and approved the final version of the manuscript.

## Funding

This work was supported by the grant no. 2012/07/E/NZ9/00194 from the National Science Centre, Poland to AB-Z.

## Conflict of Interest

The authors declare that the research was conducted in the absence of any commercial or financial relationships that could be construed as a potential conflict of interest.

## Supplementary Material

Supplemental Table S1 Gene Ontology terms for genes differentially expressed in pioneer roots identified using DAVID.

Supplemental Table S2 Gene Ontology terms for genes differentially expressed in stems identified using DAVID.

Supplemental Table S3 Gene Ontology categories over-represented in pioneer roots identified using PANTHER.

Supplemental Table S4 Gene Ontology categories over-represented in stems identified using PANTHER.
