# Supplementary material for: Xylem Cell Wall Formation in Pioneer Roots and Stems of Populus trichocarpa (Torr. & Gray)
Source: Front Plant Sci. 2019 Nov 12;10:1419. doi: 10.3389/fpls.2019.01419 (PMC6861220; doi:10.3389/fpls.2019.01419)
Supplement: Supplemental Table S1 — Gene Ontology terms for genes differentially expressed in pioneer roots identified using DAVID. [file DataSheet_1.pdf]

Supplemental Table S1

GO	Category	Term	Count	%	P-Value	Benjamini
GO:0005975	GOTERM_BP_DIRECT	carbohydrate metabolic process	29	2,1	2,9E-2	4,8E-1
GO:0071555	GOTERM_BP_DIRECT	cell wall organization	27	1,9	1,6E-8	6,3E-6
GO:0051603	GOTERM_BP_DIRECT	proteolysis involved in cellular protein catabolic process	16	1,2	1,7E-5	3,3E-3
GO:0046916	GOTERM_BP_DIRECT	cellular transition metal ion homeostasis	12	0,9	6,0E-3	2,3E-1
GO:0030001	GOTERM_BP_DIRECT	metal ion transport	12	0,9	1,4E-2	3,8E-1
GO:0030244	GOTERM_BP_DIRECT	cellulose biosynthetic process	11	0,8	7,3E-5	7,1E-3
GO:0045490	GOTERM_BP_DIRECT	pectin catabolic process	11	0,8	2,2E-2	4,4E-1
GO:0071554	GOTERM_BP_DIRECT	cell wall organization or biogenesis	10	0,7	7,1E-4	3,9E-2
GO:0030163	GOTERM_BP_DIRECT	protein catabolic process	10	0,7	1,8E-2	4,5E-1
	GOTERM_BP_DIRECT	xyloglucan metabolic process	9	0,6	8,9E-5	6,9E-3
	GOTERM_BP_DIRECT	cell wall biogenesis	9	0,6	1,1E-4	7,0E-3
	GOTERM_BP_DIRECT	auxin-activated signaling pathway	9	0,6	2,4E-2	4,5E-1
	GOTERM_BP_DIRECT	biosynthetic process	9	0,6	5,8E-2	6,9E-1
	GOTERM_BP_DIRECT	plant-type primary cell wall biogenesis	8	0,6	4,6E-5	5,9E-3
	GOTERM_BP_DIRECT	cell wall modification	8	0,6	7,5E-2	7,3E-1
	GOTERM_BP_DIRECT	oxalate metabolic process	7	0,5	2,9E-2	4,7E-1
	GOTERM_BP_DIRECT	amino acid transmembrane transport	7	0,5	9,7E-2	7,8E-1
	GOTERM_BP_DIRECT	cellulose catabolic process	6	0,4	4,2E-3	1,8E-1
	GOTERM_BP_DIRECT	ribosomal large subunit biogenesis	5	0,4	5,1E-2	6,5E-1
	GOTERM_BP_DIRECT	glycolytic process	5	0,4	6,4E-2	7,1E-1
	GOTERM_BP_DIRECT	cellular amino acid metabolic process	5	0,4	7,5E-2	7,2E-1
	GOTERM_BP_DIRECT	plant-type secondary cell wall biogenesis	4	0,3	1,3E-2	3,9E-1
	GOTERM_BP_DIRECT	xylan biosynthetic process	4	0,3	1,9E-2	4,4E-1
	GOTERM_BP_DIRECT	rRNA modification	3	0,2	2,1E-2	4,4E-1
	GOTERM_BP_DIRECT	response to freezing	3	0,2	6,6E-2	7,0E-1
	GOTERM_BP_DIRECT	regulation of response to salt stress	2	0,1	7,7E-2	7,1E-1
	GOTERM_BP_DIRECT	nitric oxide biosynthetic process	2	0,1	7,7E-2	7,1E-1
	GOTERM_BP_DIRECT	box H/ACA snoRNA 3'-end processing	2	0,1	7,7E-2	7,1E-1

GO:0016021	GOTERM_CC_DIRECT	integral component of membrane	327	23,6	1,6E-2	2,5E-1
GO:0005886	GOTERM_CC_DIRECT	plasma membrane	63	4,5	3,6E-2	3,4E-1
GO:0005618	GOTERM_CC_DIRECT	cell wall	35	2,5	1,8E-7	2,2E-5
GO:0016020	GOTERM_CC_DIRECT	membrane	35	2,5	8,8E-2	4,9E-1
	GOTERM_CC_DIRECT	extracellular region	32	2,3	1,1E-5	6,7E-4
	GOTERM_CC_DIRECT	Golgi apparatus	24	1,7	1,7E-5	6,8E-4
	GOTERM_CC_DIRECT	plant-type cell wall	16	1,2	1,7E-4	5,3E-3
	GOTERM_CC_DIRECT	anchored component of plasma membrane	14	1,0	5,4E-2	3,9E-1
	GOTERM_CC_DIRECT	apoplast	13	0,9	5,0E-4	1,2E-2
	GOTERM_CC_DIRECT	microtubule	9	0,6	4,7E-2	3,7E-1
	GOTERM_CC_DIRECT	cell periphery	8	0,6	2,4E-2	2,8E-1
	GOTERM_CC_DIRECT	vacuole	8	0,6	7,4E-2	4,7E-1
	GOTERM_CC_DIRECT	lysosome	7	0,5	9,9E-3	1,8E-1
	GOTERM_CC_DIRECT	small-subunit processome	7	0,5	3,6E-2	3,6E-1
	GOTERM_CC_DIRECT	extracellular space	7	0,5	4,4E-2	3,7E-1
	GOTERM_CC_DIRECT	phosphopyruvate hydratase complex	3	0,2	2,0E-2	2,7E-1
	GOTERM_CC_DIRECT	box C/D snoRNP complex	3	0,2	7,6E-2	4,5E-1
GO:0046872	GOTERM_MF_DIRECT	metal ion binding	53	3,8	2,5E-2	5,1E-1
GO:0016787	GOTERM_MF_DIRECT	hydrolase activity	22	1,6	6,1E-2	6,2E-1
GO:0005215	GOTERM_MF_DIRECT	transporter activity	17	1,2	6,0E-3	2,6E-1
GO:0004553	GOTERM_MF_DIRECT	hydrolase activity, hydrolyzing O-glycosyl compounds	16	1,2	1,4E-2	4,2E-1
GO:0016709	GOTERM_MF_DIRECT	oxidoreductase activity, acting on paired donors, with in	14	1,0	6,7E-2	6,1E-1
GO:0046914	GOTERM_MF_DIRECT	transition metal ion binding	12	0,9	5,6E-3	2,8E-1
GO:0016759	GOTERM_MF_DIRECT	cellulose synthase activity	10	0,7	5,7E-7	2,0E-4
GO:0016760	GOTERM_MF_DIRECT	cellulose synthase (UDP-forming) activity	10	0,7	4,1E-5	7,1E-3
GO:0016413	GOTERM_MF_DIRECT	O-acetyltransferase activity	10	0,7	5,2E-4	4,5E-2
	GOTERM_MF_DIRECT	xyloglucan:xyloglucosyl transferase activity	9	0,6	1,1E-4	1,3E-2
	GOTERM_MF_DIRECT	serine-type carboxypeptidase activity	9	0,6	2,3E-3	1,5E-1
	GOTERM_MF_DIRECT	aspartic-type endopeptidase activity	9	0,6	5,6E-2	6,1E-1
	GOTERM_MF_DIRECT	pectinesterase inhibitor activity	8	0,6	1,9E-2	4,9E-1
	GOTERM_MF_DIRECT	amino acid transmembrane transporter activity	8	0,6	5,2E-2	6,3E-1
	GOTERM_MF_DIRECT	aspartyl esterase activity	8	0,6	6,7E-2	6,2E-1

GOTERM_MF_DIRECT	oxalate decarboxylase activity	7	0,5	2,6E-2	5,0E-1
GOTERM_MF_DIRECT	cysteine-type endopeptidase activity	7	0,5	2,8E-2	5,0E-1
GOTERM_MF_DIRECT	signal transducer activity	7	0,5	4,1E-2	5,8E-1
GOTERM_MF_DIRECT	manganese ion binding	7	0,5	5,0E-2	6,3E-1
GOTERM_MF_DIRECT	nutrient reservoir activity	7	0,5	7,6E-2	6,1E-1
GOTERM_MF_DIRECT	cellulase activity	6	0,4	7,7E-3	2,8E-1
GOTERM_MF_DIRECT	calcium-dependent phospholipid binding	6	0,4	3,2E-2	5,3E-1
GOTERM_MF_DIRECT	beta-glucosidase activity	6	0,4	8,6E-2	6,5E-1
GOTERM_MF_DIRECT	microtubule binding	5	0,4	5,5E-2	6,3E-1
GOTERM_MF_DIRECT	alpha-L-arabinofuranosidase activity	4	0,3	3,4E-2	5,2E-1
GOTERM_MF_DIRECT	transaminase activity	4	0,3	6,1E-2	6,3E-1
GOTERM_MF_DIRECT	phosphopyruvate hydratase activity	3	0,2	2,0E-2	4,6E-1
GOTERM_MF_DIRECT	pectin acetylesterase activity	3	0,2	6,3E-2	6,1E-1
GOTERM_MF_DIRECT	malate dehydrogenase (decarboxylating) (NAD+) activity	3	0,2	7,4E-2	6,3E-1
GOTERM_MF_DIRECT	xylan 1,4-beta-xylosidase activity	3	0,2	9,7E-2	6,8E-1
GOTERM_MF_DIRECT	molybdopterin cofactor binding	2	0,1	7,5E-2	6,2E-1
GOTERM_MF_DIRECT	nitrate reductase (NADH) activity	2	0,1	7,5E-2	6,2E-1

Supplemental Table S2

GO	Category	Term	Count	%	P-Value	Benjamini
GO:0006355	GOTERM_BP_DIRECT	regulation of transcription, DNA-templated	36	4,0	6,2E-2	8,4E-1
GO:0005975	GOTERM_BP_DIRECT	carbohydrate metabolic process	22	2,5	1,4E-2	7,8E-1
GO:0052696	GOTERM_BP_DIRECT	flavonoid glucuronidation	12	1,3	3,2E-2	8,7E-1
GO:0009813	GOTERM_BP_DIRECT	flavonoid biosynthetic process	12	1,3	4,0E-2	8,8E-1
GO:0015979	GOTERM_BP_DIRECT	photosynthesis	10	1,1	2,0E-3	4,6E-1
GO:0055085	GOTERM_BP_DIRECT	transmembrane transport	10	1,1	4,9E-2	8,6E-1
GO:0006855	GOTERM_BP_DIRECT	drug transmembrane transport	8	0,9	4,5E-3	5,1E-1
GO:0009416	GOTERM_BP_DIRECT	response to light stimulus	5	0,6	5,4E-2	8,6E-1
	GOTERM_BP_DIRECT	response to biotic stimulus	5	0,6	8,3E-2	8,8E-1
GO:0009768	GOTERM_BP_DIRECT	photosynthesis, light harvesting in photosystem I	4	0,4	2,0E-2	7,9E-1
	GOTERM_BP_DIRECT	chaperone-mediated protein folding	4	0,4	5,5E-2	8,3E-1
	GOTERM_BP_DIRECT	protein-chromophore linkage	4	0,4	8,1E-2	8,9E-1
	GOTERM_BP_DIRECT	base-excision repair	4	0,4	8,6E-2	8,7E-1
	GOTERM_BP_DIRECT	suberin biosynthetic process	3	0,3	4,2E-2	8,5E-1
	GOTERM_BP_DIRECT	long-chain fatty-acyl-CoA metabolic process	3	0,3	4,2E-2	8,5E-1
GO:0005634	GOTERM_CC_DIRECT	nucleus	85	9,5	2,7E-2	3,5E-1
GO:0043231	GOTERM_CC_DIRECT	intracellular membrane-bounded organelle	16	1,8	1,8E-2	3,3E-1
GO:0005840	GOTERM_CC_DIRECT	ribosome	10	1,1	1,3E-2	4,5E-1
	GOTERM_CC_DIRECT	photosystem I	7	0,8	9,8E-4	8,6E-2
	GOTERM_CC_DIRECT	chloroplast envelope	6	0,7	7,4E-2	6,3E-1
	GOTERM_CC_DIRECT	photosystem II	5	0,6	2,3E-2	3,4E-1
	GOTERM_CC_DIRECT	plastoglobule	4	0,4	8,9E-2	6,1E-1
	GOTERM_CC_DIRECT	photosystem I reaction center	3	0,3	1,5E-2	3,8E-1
	GOTERM_CC_DIRECT	chloroplast outer membrane	3	0,3	7,8E-2	6,1E-1
GO:0003677	GOTERM_MF_DIRECT	DNA binding	77	8,6	1,3E-6	3,4E-4
GO:0046872	GOTERM_MF_DIRECT	metal ion binding	36	4,0	5,4E-2	6,8E-1

GO:0043565	GOTERM_MF_DIRECT	sequence-specific DNA binding	19	2,1	8,1E-2	7,3E-1
GO:0080044	GOTERM_MF_DIRECT	quercetin 7-O-glucosyltransferase activity	11	1,2	3,6E-2	7,0E-1
GO:0080043	GOTERM_MF_DIRECT	quercetin 3-O-glucosyltransferase activity	11	1,2	3,6E-2	7,0E-1
GO:0015297	GOTERM_MF_DIRECT	antiporter activity	10	1,1	1,6E-3	1,9E-1
GO:0042626	GOTERM_MF_DIRECT	ATPase activity, coupled to transmembrane movement o	10	1,1	2,5E-2	6,8E-1
	GOTERM_MF_DIRECT	serine-type endopeptidase activity	9	1,0	6,5E-2	7,2E-1
	GOTERM_MF_DIRECT	drug transmembrane transporter activity	8	0,9	4,5E-3	3,3E-1
	GOTERM_MF_DIRECT	dioxygenase activity	8	0,9	4,4E-2	6,9E-1
	GOTERM_MF_DIRECT	peptidyl-prolyl cis-trans isomerase activity	7	0,8	2,5E-2	7,3E-1
	GOTERM_MF_DIRECT	transcriptional activator activity, RNA polymerase II trans	6	0,7	7,3E-2	7,4E-1
	GOTERM_MF_DIRECT	amino acid transmembrane transporter activity	6	0,7	7,6E-2	7,3E-1
	GOTERM_MF_DIRECT	core promoter sequence-specific DNA binding	5	0,6	2,7E-2	6,5E-1
	GOTERM_MF_DIRECT	chlorophyll binding	5	0,6	3,8E-2	6,8E-1
	GOTERM_MF_DIRECT	pigment binding	4	0,4	2,0E-2	7,3E-1
	GOTERM_MF_DIRECT	FK506 binding	4	0,4	5,1E-2	6,8E-1
	GOTERM_MF_DIRECT	fatty-acyl-CoA reductase (alcohol-forming) activity	3	0,3	4,8E-2	6,9E-1

Supplemental Table S3

Analysis Type:	PANTHER Overrepresentation Test (release 20160715)		Reference list	Client Text Box Input	
Annotation Version and Release Date:	GO Ontology database Released 2017-02-28	Mapped IDs:	41462 out of 41462	1260 out of 1260	
Analyzed List:	Client Text Box Input (Populus trichocarpa)	Unmapped IDs:	0	147	
Reference List:	Populus trichocarpa (all genes in database)	Multiple mapping information:	0	0	
Bonferroni correction:	true				
Bonferroni count:	1494				

**GO biological process complete**	Populus trichocarpa - REFLIST (41462)	Client Text Box Input (1260)	Client Text Box Input (expected)	Client Text Box Input (over/under)	Client Text Box Input (fold Enrichment)	Client Text Box Input (P-value)
mRNA transcription (GO:0009299)	20	7	0,61	+	11,52	5,27E-03
plant-type primary cell wall biogenesis (GO:0009833)	29	10	0,88	+	11,35	5,08E-05
cytoskeleton-dependent cytokinesis (GO:0061640)	31	10	0,94	+	10,61	9,38E-05

mitotic cytokinesis (GO:0000281)	31	10	0,94	+	10,61	9,38E-05
cytokinesis (GO:0000910)	43	12	1,31	+	9,18	2,23E-05
plant-type cell wall biogenesis (GO:0009832)	72	15	2,19	+	6,86	1,76E-05
xyloglucan metabolic process (GO:0010411)	53	11	1,61	+	6,83	1,58E-03
cell wall biogenesis (GO:0042546)	122	25	3,71	+	6,74	3,90E-10
cell wall polysaccharide metabolic process (GO:0010383)	75	13	2,28	+	5,7	1,26E-03
hemicellulose metabolic process (GO:0010410)	75	13	2,28	+	5,7	1,26E-03
cellulose biosynthetic process (GO:0030244)	58	10	1,76	+	5,67	2,37E-02
cell division (GO:0051301)	85	13	2,58	+	5,03	4,85E-03
cell wall macromolecule metabolic process (GO:0044036)	116	17	3,53	+	4,82	2,87E-04
cellulose metabolic process (GO:0030243)	86	12	2,61	+	4,59	2,80E-02
cellular glucan metabolic process (GO:0006073)	194	23	5,9	+	3,9	9,89E-05
cellular polysaccharide metabolic process (GO:0044264)	230	27	6,99	+	3,86	9,07E-06

plant-type cell wall organization or biogenesis (GO:0071669)	168	19	5,11	+	3,72	2,62E-03
glucan metabolic process (GO:0044042)	204	23	6,2	+	3,71	2,37E-04
cell wall organization or biogenesis (GO:0071554)	539	60	16,38	+	3,66	6,35E-14
cell wall organization (GO:0071555)	385	41	11,7	+	3,5	2,28E-08
external encapsulating structure organization (GO:0045229)	388	41	11,79	+	3,48	2,87E-08
polysaccharide biosynthetic process (GO:0000271)	167	17	5,08	+	3,35	3,38E-02
polysaccharide metabolic process (GO:0005976)	412	41	12,52	+	3,27	1,69E-07
cellular carbohydrate metabolic process (GO:0044262)	348	34	10,58	+	3,21	9,96E-06
chemical homeostasis (GO:0048878)	375	32	11,4	+	2,81	5,36E-04
ion homeostasis (GO:0050801)	262	22	7,96	+	2,76	4,32E-02
carbohydrate metabolic process (GO:0005975)	1187	83	36,07	+	2,3	1,01E-08
biological_process (GO:0008150)	20829	711	632,98	+	1,12	9,11E-03

Unclassified (UNCLASSIFIED)	20633	549	627,02	-	0,88	0,00E+00
nucleic acid phosphodiester bond hydrolysis (GO:0090305)	801	6	24,34	-	0,25	1,32E-02

Supplemental Table S4

Analysis Type:	PANTHER Overrepresentation Test (release 20160715)			Reference list	Client Text Box Input	
Annotation Version and Release Date:	GO Ontology database Released 2017-02-28		Mapped IDs:	41462 out of 41462	821 out of 821	
Analyzed List:	Client Text Box Input (Populus trichocarpa)		Unmapped IDs:	0	86	
Reference List:	Populus trichocarpa (all genes in database)		Multiple mapping information:	0	0	
Bonferroni correction:	true					
Bonferroni count:	1494					
**GO biological process complete**	Populus trichocarpa - REFLIST (41462)	Client Text Box Input (821)	Client Text Box Input (expected)	Client Text Box Input (over/under)	Client Text Box Input (fold Enrichment)	Client Text Box Input (P-value)
biological_process (GO:0008150)	20829	490	412,44	+	1,19	0,0000509
cell differentiation (GO:0030154)	293	23	5,8	+	3,96	0,0000702
cellular developmental process (GO:0048869)	314	23	6,22	+	3,7	0,000235
regulation of biosynthetic process (GO:0009889)	2654	90	52,55	+	1,71	0,000986
regulation of cellular biosynthetic process (GO:0031326)	2654	90	52,55	+	1,71	0,000986

regulation of cellular macromolecule biosynthetic process (GO:2000112)	2607	90	51,62	+	1,74	0,000468
regulation of cellular metabolic process (GO:0031323)	2881	93	57,05	+	1,63	0,00484
regulation of gene expression (GO:0010468)	2790	92	55,25	+	1,67	0,00234
regulation of macromolecule biosynthetic process (GO:0010556)	2651	90	52,49	+	1,71	0,000941
regulation of macromolecule metabolic process (GO:0060255)	3071	96	60,81	+	1,58	0,0121
regulation of metabolic process (GO:0019222)	3103	96	61,44	+	1,56	0,0182
regulation of nitrogen compound metabolic process (GO:0051171)	2681	91	53,09	+	1,71	0,000817
regulation of nucleic acid-templated transcription (GO:1903506)	2524	89	49,98	+	1,78	0,000224
regulation of nucleobase-containing compound metabolic process (GO:0019219)	2568	91	50,85	+	1,79	0,000129
regulation of primary metabolic process (GO:0080090)	2896	94	57,34	+	1,64	0,00341

regulation of RNA biosynthetic process (GO:2001141)	2524	89	49,98	+	1,78	0,000224
regulation of RNA metabolic process (GO:0051252)	2543	90	50,35	+	1,79	0,000162
regulation of transcription, DNA-templated (GO:0006355)	2484	89	49,19	+	1,81	0,000113
single-organism developmental process (GO:0044767)	603	30	11,94	+	2,51	0,00991
Unclassified (UNCLASSIFIED)	20633	331	408,56	-	0,81	0
